# GABA_B_ receptor upregulates fragile X mental retardation protein expression in neurons

**DOI:** 10.1038/srep10468

**Published:** 2015-05-28

**Authors:** Wenhua Zhang, Chanjuan Xu, Haijun Tu, Yunyun Wang, Qian Sun, Ping Hu, Yongjian Hu, Philippe Rondard, Jianfeng Liu

**Affiliations:** 1 Cellular Signaling Laboratory, Key Laboratory of Molecular Biophysics of Ministry of Education, College of Life Science and Technology and the Collaborative Innovation Center for Brain Science, Huazhong University of Science and Technology, Wuhan, Hubei, China; 2 Institut de Génomique Fonctionnelle, CNRS UMR5203, INSERM U1191, Université de Montpellier, Montpellier, France

## Abstract

Fragile X mental retardation protein (FMRP) is an RNA-binding protein important for the control of translation and synaptic function. The mutation or silencing of FMRP causes Fragile X syndrome (FXS), which leads to intellectual disability and social impairment. γ-aminobutyric acid (GABA) is the major inhibitory neurotransmitter of the mammalian central nervous system, and its metabotropic GABA_B_ receptor has been implicated in various mental disorders. The GABA_B_ receptor agonist baclofen has been shown to improve FXS symptoms in a mouse model and in human patients, but the signaling events linking the GABA_B_ receptor and FMRP are unknown. In this study, we found that GABA_B_ receptor activation upregulated cAMP response element binding protein-dependent Fmrp expression in cultured mouse cerebellar granule neurons via two distinct mechanisms: the transactivation of insulin-like growth factor-1 receptor and activation of protein kinase C. In addition, a positive allosteric modulator of the GABA_B_ receptor, CGP7930, stimulated Fmrp expression in neurons. These results suggest a role for GABA_B_ receptor in Fmrp regulation and a potential interest of GABA_B_ receptor signaling in FXS improvement.

Fragile X mental retardation protein (FMRP) is an RNA-binding protein that controls translation and synaptic function^1^,^2^. FMRP mutation or silencing causes Fragile X syndrome (FXS), a common inherited disease associated with autism, intellectual disability, and social impairment^3^. Chemical compounds targeting metabotropic glutamate receptor 5 (mGluR5) and other neurotransmitter receptors such as γ-aminobutyric acid and serotonin receptors^4^,^5^ or downstream signaling pathways such as mitogen-activated protein kinase kinase (MEK)/extracellular signal-regulated kinase (ERK)1/2 and phosphatidylinositol 3 kinase (PI3K)/glycogen synthase kinase 3β/Akt^6^ have been tested for their ability to improve FXS symptoms such as anxiety, seizure, and hyperactivity. Recent studies have demonstrated that the GABA_B_ receptor agonist R-baclofen (STX209) can improve locomotor activity and motor coordination in patients with FXS and modify the pathophysiology induced by FMRP deficiency including the effects on protein synthesis, AMPA receptor turnover, and dendritic spine density^7^,^8^, suggesting a connection between GABA_B_ receptor and FMRP regulation. However, the signaling events linking GABA_B_ receptor activation to FMRP are not well understood.

The GABA_B_ receptor is the metabotropic receptor of GABA, the main inhibitory neurotransmitter in the mammalian central nervous system^9^. The receptor is a seven transmembrane domain-containing protein belonging to class C G protein-coupled receptors (GPCRs)^10^ and is assembled as a heterodimer containing GABA_B1_ and GABA_B2_ subunits^9^. Only GABA_B1_ subunit can bind agonists, whereas GABA_B2_ subunit is responsible for G protein coupling^11^. Positive allosteric modulators bind within the GABA_B2_ transmembrane domain to potentiate the effect of agonists^12^. Presynaptic GABA_B_ receptor activation inhibits neurotransmitter release through the depression of voltage-gated Ca^2+^ channels, whereas activated postsynaptic GABA_B_ receptors open K^+^ channels to induce neuronal hyperpolarisation^13^. GABA_B_ receptor activation induces the ERK_1/2_/cAMP response element-binding protein (CREB) signaling pathway, which is dependent on G_i/o_ protein^14^. GABA_B_ receptor also activates PI3K/Akt signaling to decrease apoptosis in cerebellar granule neurons (CGNs)^15^,^16^ through transactivation of the insulin-like growth factor-1 receptor (IGF-1R).

This study investigated the link between GABA_B_ receptor and Fmrp. The results show that activation of the GABA_B_ receptor upregulated *Fmr1* mRNA and protein expression via activation of CREB. IGF-1R- and protein kinase C (PKC)-dependent signaling pathways were found to be involved in CREB activation and Fmrp synthesis. In addition, we show that CGP7930, a positive allosteric modulators (PAMs) of GABA_B_ receptor, also upregulated Fmrp expression.

## Results

### GABA_B_ receptor activation upregulates Fmrp expression

**Figure 1:**
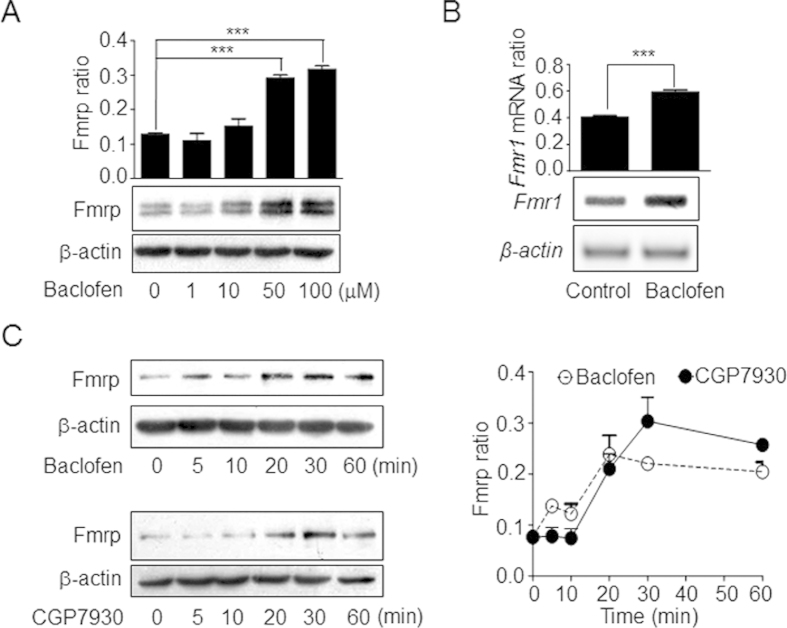
Activation of GABA_B_ receptor upregulates Fmrp expression in CGNs. **(A)** Fmrp expression in CGNs treated with indicated concentrations of baclofen. ***P < 0.001 vs. basal levels. **(B)** *Fmr1* mRNA expression upon treatment with baclofen. **(C)** Time course of Fmrp expression induced by baclofen and CGP7930. Fmrp expression level was quantified based on three independent experiments (mean ± SEM). ***P < 0.001 vs. basal levels. Fmrp ratio and *Fmr1* mRNA ratio were defined as the ratio between the density of each band and the sum of the densities of all the bands in a given blot. Full-size blots are shown in Supplementary Figure S1 and the band of interest is indicated by a red box.

The link between GABA_B_ receptor activation and Fmrp expression was investigated using the GABA_B_ receptor agonist baclofen. Drug treatment increased Fmrp level in a dose-dependent manner in CGNs (Fig. 1A, Figure S1A), and increased *Fmr1* mRNA expression as well as Fmrp protein synthesis in a time-dependent manner (Fig. 1B, C **upper panel, Figure S1B, Figure S2**) starting 20 min after drug application, with effects persisting for more than 60 min.

PAMs potentiate the GABA_B_ receptor activation by orthosteric agonists such as baclofen^17^; in the case of CGP7930, this is accomplished via binding to the transmembrane domain of GABA_B2_ subunit^18^,^19^,^20^. We recently showed that CGP7930 can directly activate the GABA_B_ receptor in cultured cell lines and neurons in the absence of an agonist^14^,^15^. We found here that the kinetics of Fmrp expression after CGP7930 treatment were similar to those induced by baclofen (Fig. 1C, Figure S1C). These data demonstrate that GABA_B_ receptor activation via the GABA_B2_ subunit increases Fmrp expression.

### CREB is required for Fmrp upregulation induced by GABA_B_ receptor

**Figure 2:**
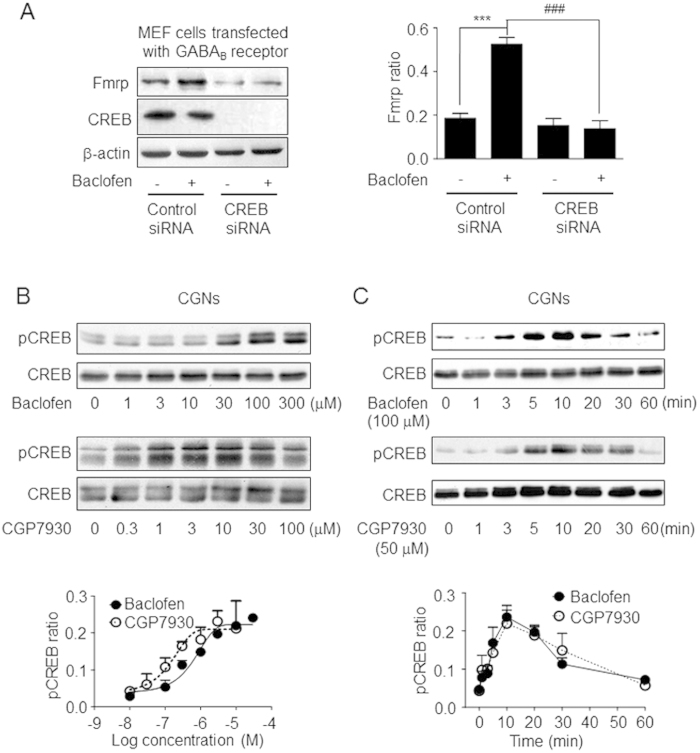
GABA_B_ receptor-induced CREB signaling is required for Fmrp upregulation. **(A)** Effect of siRNA-mediated knockdown of CREB on baclofen-induced Fmrp expression in MEFs co-transfected with GABA_B1_ and GABA_B2_. Representative western blots are shown. CREB phosphorylation level in control siRNA-transfected cells. Fmrp ratio was defined as in Fig. 1A. The level in baclofen-treated cells was quantified based on three independent experiments (mean ± SEM). ***P < 0.001 vs. basal with control siRNA. ^###^P < 0.001 vs. baclofen-treated cells transfected with control siRNA. **(B)** CREB phosphorylation in CGNs induced by indicated concentrations of baclofen or CGP7930. The data were quantified from three independent experiments (mean ± SEM). **(C)** Time course of CREB phosphorylation induced by baclofen and CGP7930. Protein level was quantified based on three independent experiments (mean ± SEM). pCREB ratio was defined as the ratio between the density of each band and the sum of the densities of all the bands in a given blot. Full-size blots are shown in Supplementary Figure S3 and the band of interest is indicated by a red box.

The *Fmr1* gene promoter contains a CREB-binding site, and mGluR1 and 5 can regulate Fmrp expression through CREB^21^,^22^. Moreover, CREB itself is regulated by various receptors via downstream effectors such as PKA, PKC, ERK_1/2_, and Akt^23^,^24^,^25^. The role of CREB in GABA_B_ receptor-mediated Fmrp upregulation was investigated by short interfering (si)RNA-knockdown of CREB in mouse embryonic fibroblasts (MEFs) co-transfected with GABA_B1_ and GABA_B2_. CREB depletion abolished the GABA_B_ receptor-induced increase in Fmrp expression relative to the control (Fig. 2A, Figure S3A). These data indicate that GABA_B_ receptor-induced CREB activity is required for Fmrp synthesis.

In CGNs, baclofen and CGP7930 treatment induced a concentration-dependent increase in the level of phosphorylated CREB without altering total CREB expression level (Fig. 2B, Figure S3B). The rapid and transient increase in CREB phosphorylation peaked at 10 min and decreased to the basal level at 60 min after drug application (Fig. 2C, **Figure S3C**). Interestingly, pre-treatment of CGNs with the competitive GABA_B_ receptor antagonist CGP54626 blocked baclofen but not CGP7930-induced CREB phosphorylation (Figure S4). These results indicate that GABA_B_ receptor activation can induce a transient increase in phosphorylation of CREB, a component of signaling pathway that important for Fmrp expression.

### GABA_B_ receptor-mediated transactivation of IGF-1R leads to CREB activation

**Figure 3:**
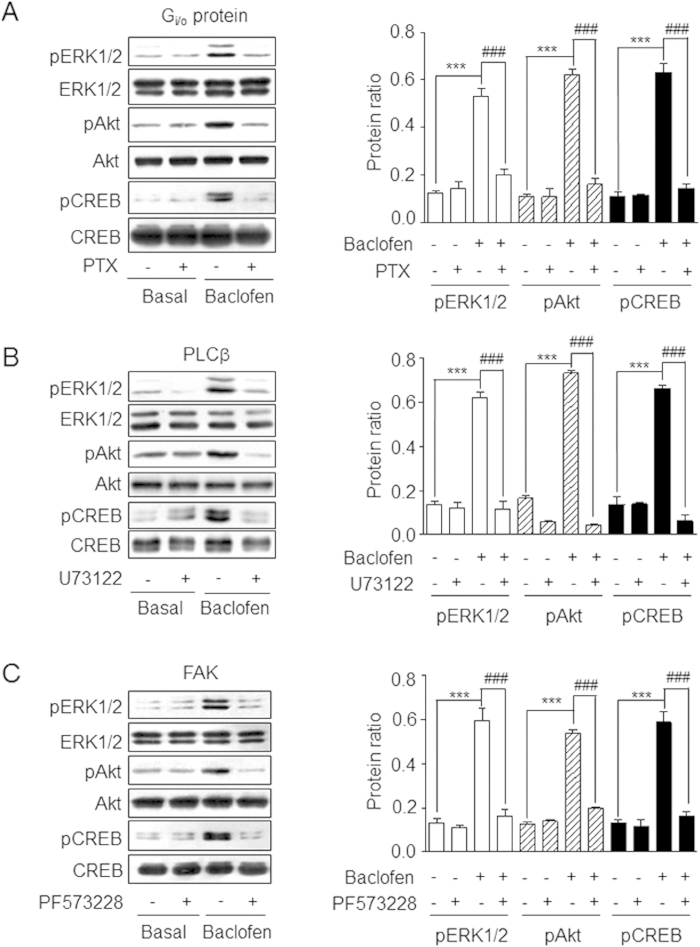
GABA_B_ receptor-mediated CREB phosphorylation is dependent on G_i/o_ protein, PLCβ, and FAK. CGNs were pretreated with PTX **(A)**, U73122 **(B)**, or PF573228 **(C)** before baclofen-stimulation. CREB, ERK_1/2_ and Akt phosphorylation was detected by western blotting. Protein ratio on the Y-axis was defined as the ratio between the density of each band and the sum of the densities of all the bands in a given blot. Data represent the mean ± SEM from three separate sets of immunoblots. ***P < 0.001 vs. basal level. ^###^P < 0.001 vs. baclofen-treated group. Full-size blots are shown in Supplementary Figure S5 and the band of interest is indicated by a red box.

**Figure 4:**
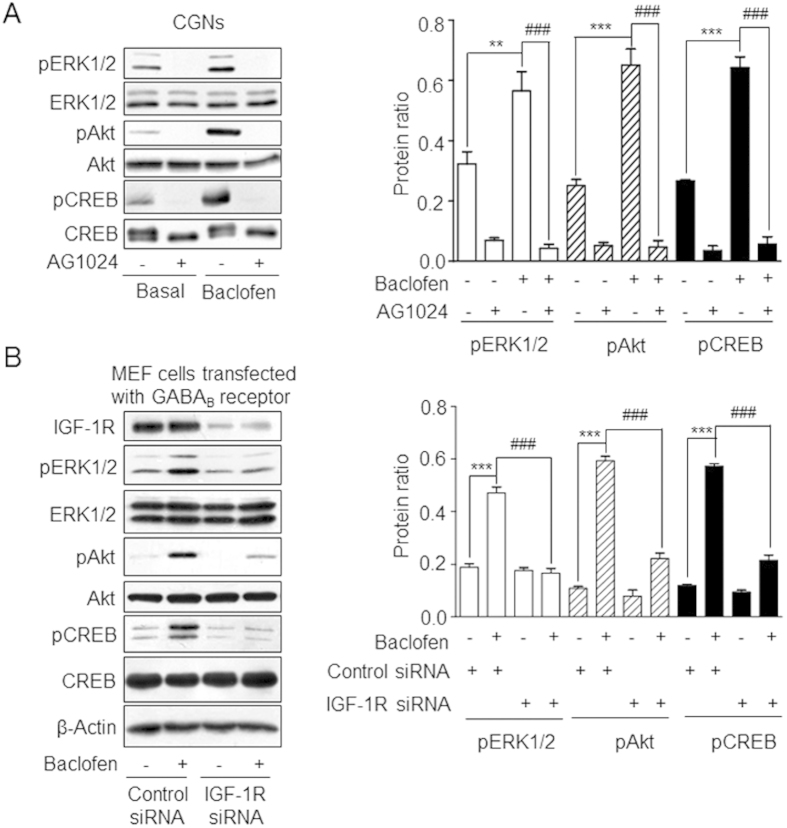
GABA_B_ receptor-mediated transactivation of IGF-1R is required for CREB phosphorylation. **(A)** CGNs were pre-treated with the IGF-1R inhibitor AG1024 followed by baclofen. Expression levels of pERK_1/2_, pAkt and pCREB were quantified by western blotting. Data represent the mean ± SEM from at least three independent experiments. **P < 0.01, ***P < 0.001 vs. basal level. ^###^P < 0.001 vs. baclofen-treated group. **(B)** MEFs were transfected with GABA_B1_, GABA_B2_, and control or IGF-1R siRNA before treatment with baclofen. Phosphorylation of ERK_1/2_, Akt, and CREB was quantified by western blotting. ***P < 0.001 vs. basal level in control siRNA-transfected cells. ^###^P < 0.001 vs. baclofen-treated cells transfected with control siRNA. Protein ratio was defined as in Fig. 3. Full-size blots are shown in Supplementary Figure S6 and the band of interest is indicated by a red box.

IGF-1R was reported to be transactivated by GABA_B_ receptor through G_i/o_ protein, PLCβ and focal adhesion kinase (FAK), and then further induced MEK/ERK_1/2_ and PI3K/Akt activation^15^,^16^. Therefore, we investigated the role of the IGF-1R transactivation signaling pathway in the activation of CREB. ERK_1/2_, Akt, and CREB phosphorylation were blocked by treatment with pertussis toxin (PTX), which uncouples G_i/o_ proteins from GPCRs via ADP-ribosylation of Gα_i/o_ subunits (Fig. 3A**, Figure S5A**), suggesting that GABA_B_ receptor-mediated CREB activation in CGNs is G_i/o_ protein-dependent. Pre-treatment of CGNs with U73122 (PLCβ inhibitor) or PF573228 (FAK inhibitor) blocked baclofen-induced ERK_1/2_, Akt, and CREB phosphorylation (Fig. 3B, C, **Figure S5B, C**). Furthermore, the IGF-1R inhibitor AG1024 also blocked the baclofen-induced phosphorylation of CREB in CGNs, as well as that of ERK_1/2_ and Akt (Fig. 4A, Figure S6A). In MEFs expressing the recombinant GABA_B_ receptor, siRNA knockdown of endogenous IGF-1R reduced baclofen-induced phosphorylation of CREB, ERK_1/2_, and Akt (Fig. 4B, Figure S6B). Similar results were obtained by short hairpin-mediated knockdown in MEFs using a shRNA targeting IGF-1R (Figure S7). Taken together, these results show that IGF-1R transactivation via G_i/o_ protein, PLCβ, and FAK is important for baclofen-induced CREB activation.

### PKC is required for GABA_B_ receptor-induced CREB activation independent of IGF-1R signaling

**Figure 5:**
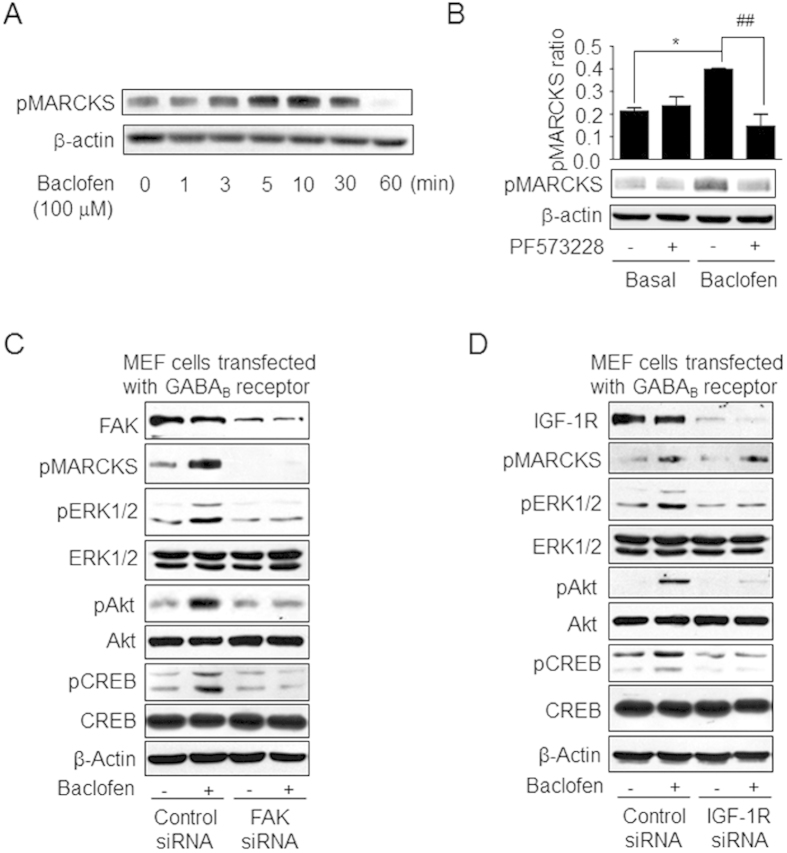
PKC activated by GABA_B_ receptor acts downstream of FAK and is independent of IGF-1R signaling. **(A)** Time course of phosphorylation of the PKC substrate MARCKS induced by baclofen in CGNs. **(B)** Effect of the FAK inhibitor PF573228 on MARCKS phosphorylation in CGNs. pMARCKS ratio was defined as the ratio between the density of each band and the sum of the densities of all the bands in a given blot. Values represent the mean ± SEM from three independent experiments. *P < 0.05 vs. basal level. ^##^P < 0.01 vs. baclofen-treated group. **(C,D)** Effect of siRNA-mediated knockdown of FAK or IGF-1R on baclofen-induced phosphorylation of MARCKS, ERK_1/2_, Akt, and CREB in MEFs co-transfected with GABA_B1_ and GABA_B2_. Full-size blots are shown in Supplementary Figure S8 and the band of interest is indicated by a red box.

**Figure 6:**
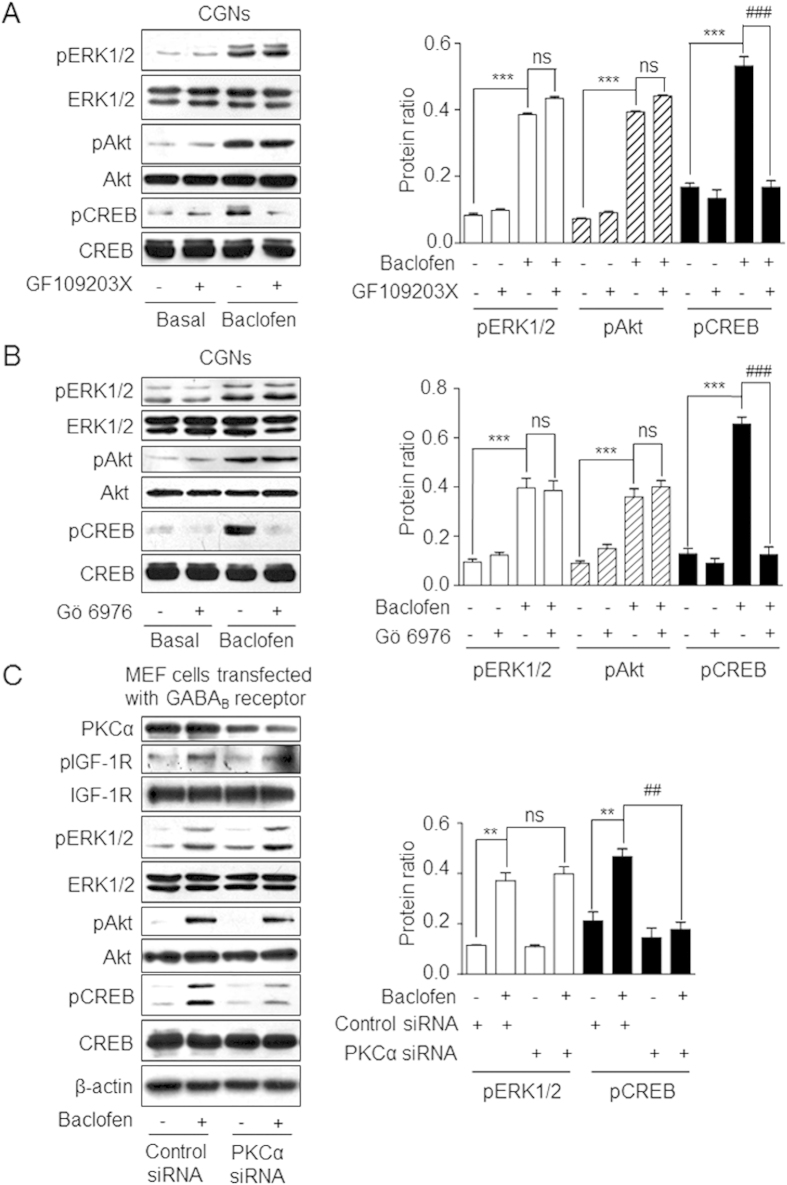
PKC is required for GABA_B_ receptor-induced CREB activation. **(A, B)** CGNs were pre-treated with GF109203x (A) or Gö-6976 (B) followed by treatment with baclofen. ERK_1/2_, Akt, and CREB phosphorylation was quantified by western blotting and the protein ratio was defined as in Fig. 3. ***P < 0.001, vs. basal level. ^###^P < 0.001, ns, not significant vs. baclofen-treated group. **(C)** MEFs were co-transfected with GABA_B1_, GABA_B2_, and control or PKCα siRNA and then treated with baclofen. pERK_1/2_ and pCREB levels were quantified by western blotting and the protein ratio was defined as in panels A and B. Data represent the mean ± SEM from three independent experiments. **P < 0.01, vs. basal level in control siRNA-transfected cells. ^##^P < 0.01, ns, not significant vs. baclofen-treated cells transfected with control siRNA. Full-size blots are shown in Supplementary Figure S9 and the band of interest is indicated by a red box.

PKC was previously shown to be activated by baclofen treatment^15^. Phosphorylation of the PKC substrate MARCKS was increased in a time-dependent manner by baclofen treatment (Fig. 5A**, Figure S8A**). Phospho-MARCKS level was reduced by application of the FAK inhibitor PF573228 or by siRNA-mediated knockdown of FAK (Fig. 5B, C, Figure S8B, C), but not by IGF-1R knockdown (Fig. 5D, Figure S8D), suggesting that PKC acts downstream of FAK but independently of IGF-1R. Three PKC inhibitors (GF109203x, Gö-6983, and Gö-6976) were used to analyse the effect of PKC on CREB activation; GF109203x and Gö-6983 inhibit all PKC isozymes^25^,^26^, whereas Gö-6976 is selective for Ca^2+^ -sensitive PKC isotypes^26^. All three inhibitors blocked baclofen-induced CREB phosphorylation but had no effect on the phosphorylation of ERK_1/2_ and Akt (Fig. 6A, B, Figure S9A, B and Figure S10A). In addition, siRNA-mediated knockdown of PKCα or PKCβ in MEFs co-expressing GABA_B1_ and GABA_B2_ subunits of the GABA_B_ receptor decreased baclofen-mediated CREB phosphorylation, whereas no changes in IGF-1R transactivation or ERK_1/2_ and Akt phosphorylation were observed (Fig. 6C**, Figure S9C** and Figure S10B). These results indicate that Ca^2+^ -sensitive PKCs are required for GABA_B_-induced CREB activation, but that this effect is independent of IGF-1R transactivation.

### IGF-1R and PKC are required for GABA_B_ receptor-induced upregulation of Fmrp expression

**Figure 7:**
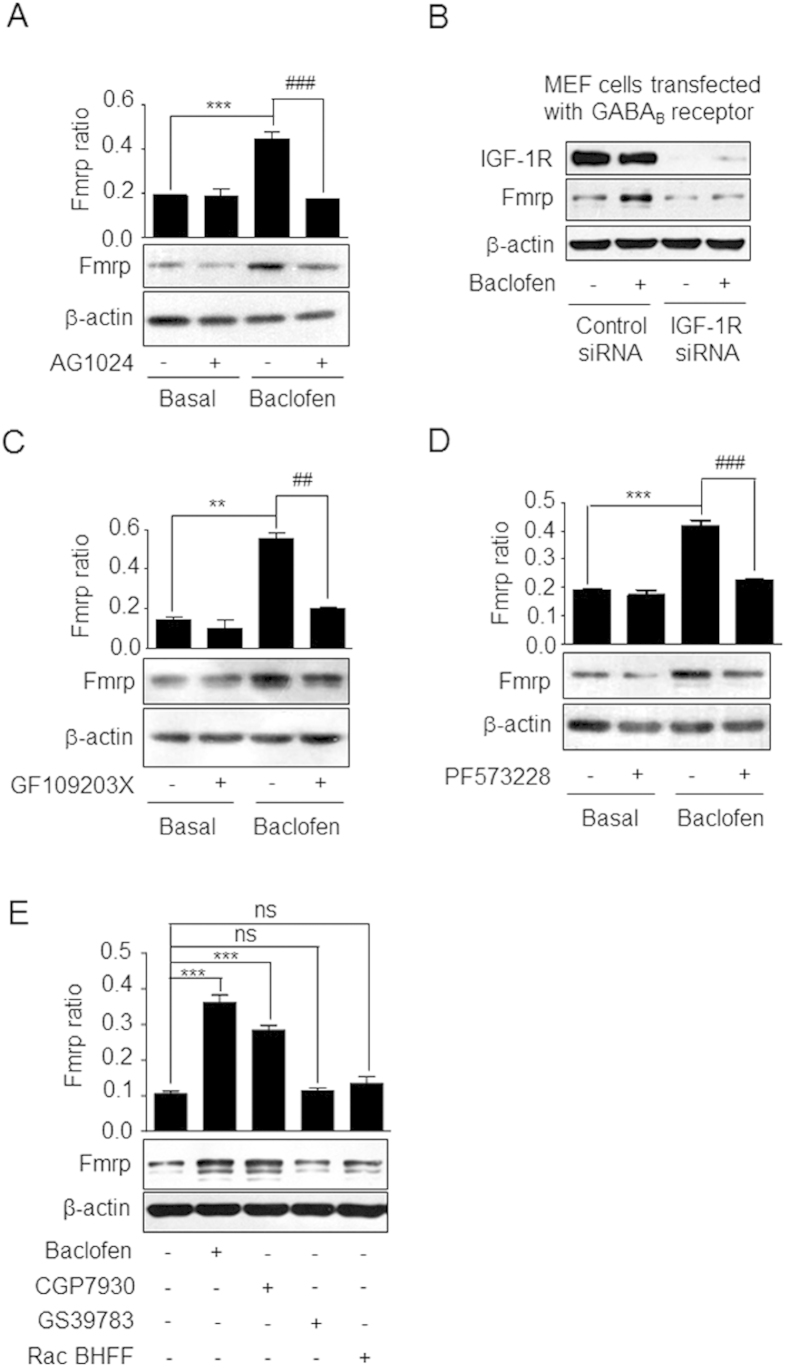
IGF-1R and PKC are involved in GABA_B_ receptor-mediated Fmrp synthesis. **(A)** CGNs were pretreated with AG1024 followed by treatment with baclofen. Fmrp levels were detected by western blotting and Fmrp ratio was defined as in Fig. 1A. Data represent the mean ± SEM from three independent experiments. **(B)** MEFs were co-transfected with GABA_B1_, GABA_B2_, and control or IGF-1R siRNA and then treated with baclofen. Fmrp levels were quantified as in panel A. **(C, D)** CGNs were pretreated with GF109203x or PF573228, and then treated with baclofen. Fmrp levels were detected as in panel A. Data represent the mean ± SEM from three independent experiments. For results in A, C, D, **P < 0.01, ***P < 0.001 vs. basal level; ^##^P < 0.01, ^###^P < 0.001, vs. baclofen-treated group. **(E)** CGNs were treated with vehicle, baclofen, CGP7930, GS39783, or Rac BHFF and Fmrp expression level was quantified as in panel A. Data represent the mean ± SEM from three independent experiments. ***P < 0.001, ns, not significant vs. basal level. Full-size blots are shown in Supplementary Figure S11 and the band of interest is indicated by a red box.

The role of IGF-1R and PKC in the GABA_B_ receptor-induced expression of Fmrp was assessed. GABA_B_ receptor-induced Fmrp synthesis was markedly reduced in CGNs by treatment with IGF-1R inhibitor (Fig. 7A, Figure S11A); siRNA-mediated IGF-1R knockdown abolished the baclofen-induced increase in Fmrp level in MEFs expressing the recombinant GABA_B_ receptor (Fig. 7B, Figure S11B). PKC inhibitor also reduced baclofen-induced Fmrp expression (Fig. 7C, Figure S11C). FAK acts upstream of IGF-1R^16^ and PKC (Fig. 5B, C) in the GABA_B_ receptor-mediated signaling pathway. Accordingly, pre-treatment with PF573228 decreased baclofen-induced Fmrp synthesis (Fig. 7D, Figure S11D). Taken together, these results indicate that both IGF-1R and PKC are critical for the upregulation of Fmrp expression induced by GABA_B_ receptor activation.

### GABA_B_ receptor PAM increases Fmrp expression

PAMs bind to the transmembrane domain of the GABA_B_ receptor at a location independent of the agonist-binding site, thereby potentiating the effect of the receptor agonist. Of the three commercially available GABA_B_ receptor PAMs (CGP7930, GS39783, and Rac BHFF), CGP7930 and Rac BHFF but not GS39783 act as PAM agonists^18^,^19^,^27^,^28^,^29^. The PAMs were compared with respect to their effects on signaling events downstream of GABA_B_ receptor activation. Interestingly, CGP7930 but not GS39783 or Rac BHFF induced the phosphorylation of Akt and CREB and increased the level of Fmrp in a manner similar to the agonist baclofen (Fig. 7E and Figure S12), confirming the role of GABA_B_ receptor activation in the modulation of Fmrp expression.

## Discussion

**Figure 8:**
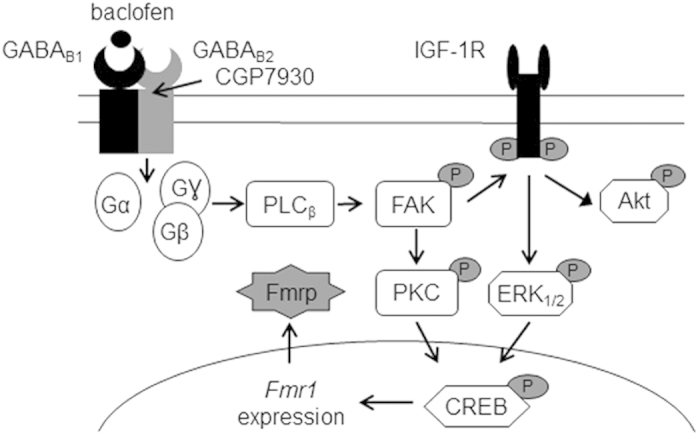
Schematic representation of the signaling pathway mediated by GABA_B_ receptor leading to CREB activation and Fmrp upregulation in CGNs. Agonist (baclofen) or PAM (CGP7930) activates the GABA_B_ receptor, leading to G_i/o_ protein/PLCβ/FAK activation, which in turn transactivates the IGF-1R signaling pathway and induces PKC-mediated CREB phosphorylation, thereby upregulating Fmrp expression both at the mRNA and protein levels.

This study investigated the signaling events linking GABA_B_ receptor activation to Fmrp expression. The results demonstrate that activation of the GABA_B_ receptor by baclofen upregulates Fmrp synthesis via induction of CREB, which involves IGF-1R- and PKC-dependent signaling (Fig. 8). We also found that the GABA_B_ receptor PAM CGP7930 upregulated Fmrp expression.

Our results clarify the signaling link between GABA_B_ receptor and Fmrp expression. However, they cannot explain the beneficial effect of baclofen in FXS mouse model or in patients, where the *Fmr1* gene is deleted or expression is blocked^4^,^30^. One possible explanation to reconcile these different findings may be through the activation of CREB by the GABA_B_ receptor. CREB is a transcription factor involved in the activation of many genes^25^; CREB phosphorylation at serine 133 promotes its binding to the CRE site and leads to gene transcription^25^ and regulates learning and memory^23^,^31^. CREB-targeted genes may facilitate memory formation through the induction of long-term potentiation or long-term depression of synaptic plasticity^32^,^33^, the growth and formation of new synaptic spines and connections^33^,^34^, or new protein synthesis participating in memory reconstruction^35^ which might help to improve cognition in FXS.

Our study suggests a novel physiological role for Frmp in neurons. Indeed, Fmrp is wildly expressed in neurons and participates in a number of intracellular processes involving mRNAs metabolism related to synaptic function and maturation^2^,^36^. Several reports also implied that Fmrp played a role in neuronal survival and apoptosis^37^,^38^; the GABA_B_ receptor was also found to transactivate IGF-1R and protect neurons against apoptosis^15^, suggesting a possible role of Fmrp in mediating the anti-apoptotic effects of GABA_B_ receptor.

IGF-1R and PKC act independently in GABA_B_ receptor/CREB/Fmrp regulation, but it is unclear how these two signaling pathways from membrane receptor and intracellular kinase integrated. We showed in our previous study that FAK serves as a platform for recruiting G protein, IGF-1R, and Akt to the activated GABA_B_ receptor and further regulating GABA_B_ receptor-induced neuroprotection^16^. FAK also has high affinity for PI3K and PLCγ^39^. PKC and its substrate MARCKS were reported to be activated after FAK phosphorylation^40^, supporting our finding that FAK acts upstream of PKC. However, as the phosphorylation profiles of FAK tyrosine and serine residues are important for distinct signal transduction cascades^39^,^41^, how FAK phosphorylation regulates IGF-1R and PKC is still under investigation. Meanwhile, given that PKC translocates from the cytosol to the membrane after GABA_B_ receptor activation to modulate desensitization^42^, the PKC pathway may play a role in controlling CREB and Fmrp activity.

PAMs bind to the GABA_B_ receptor at a site distinct from agonists such as R-baclofen (STX209)^16^,^25^,^26^, which was shown to improve FXS-associated symptoms in mice and humans^7^,^8^,^43^. Extensive data from preclinical studies on GABA_B_ receptor PAMs indicate that their benefits are similar to those of agonists, but with superior side effect profiles^44^. Among them, GS39783 was used to treat FXS mice and showed no significant improvement in an audiogenic seizure test^43^, consistent with our observation that GS39783 had no effect on CREB activation and Fmrp expression. CGP7930 and Rac BHFF, both of which exhibit PAM agonist activity^27^, have not yet been tested in an FXS mouse model. In our study, only CGP7930 induced an upregulation in the level of Fmrp expression similar to baclofen. This is consistent with the finding of CGP7930 alone being able to activate ERK_1/2_ and Akt signaling^14^,^15^. These results suggest that CGP7930 is a promising candidate for the treatment of FXS symptoms and useful for the development of novel drugs.

## Methods

### Drugs

GABA was purchased from Sigma (St. Louis, MO, USA). (R)-Baclofen, CGP54626, CGP7930, GS39783, Rac BHFF, PTX, U73122, and PF573228 were purchased from Tocris (Fisher-Bioblock, Illkirch, France). AG1024 was purchased from Santa Cruz Biotechnology (Shanghai, China). Foetal bovine serum (FBS) and other solutions used for cell culture were from Invitrogen (Shanghai, China).

### Antibodies

Primary antibodies against phospho-ERK_1/2_ (rabbit monoclonal), ERK_1/2_, phospho-Akt (Ser473) (193H12; rabbit monoclonal), Akt, phospho-CREB (rabbit), phospho-MARCKS, CREB, IGF-1Rβ, β-actin, and Fmrp, as well as horseradish peroxidase (HRP)-conjugated secondary antibodies were purchased from Cell Signaling Technology (Shanghai, China). Antibodies against PKCα and PKCβ were from Santa Cruz Biotechnology.

### Primary culture of CGNs

All animal experiments were approved by the Animal Experimentation Ethics Committee of the School of Life Science and Technology at Huazhong University of Science and Technology and were carried out in accordance with the approved guidelines for the Care and Use of Laboratory Animals of the National Institutes of Health (Bethesda, MD, USA). Primary CGN cultures were established as previously described^14^. Briefly, the cerebellum was dissected from 1-week-old KunMing mice of either sex obtained from Hubei Provincial Center for Disease Control and Prevention. Cells were maintained in a 1:1 mixture of Dulbecco’s Modified Eagle’s Medium (DMEM) with F-12 nutrients (Invitrogen) supplemented with 30 mM glucose, 2 mM glutamine, 3 mM sodium bicarbonate, 5 mM HEPES buffer, 30 mM KCl, and 10% FBS.

### MEFs culture and transfection

MEFs were cultured in DMEM supplemented with 10% FBS. For RNA interference experiments, MEFs were transfected using Lipofectamine 2000 (Thermo Fisher Scientific, Shanghai, China) according to the manufacturer’s protocol, using siRNAs against IGF-1Rα/β (sc-35638), PKCα (sc-208), PKCβII (sc-210), and FAK (sc-35353) or control siRNA-A (sc-37007) from Santa Cruz Biotechnology. One day after transfection, cells were transfected with HA-GABA_B1_ and Flag-GABA_B2_ plasmids for another 24 h before drug treatment.

### Drug treatments

Cultures were washed once with Ca^2+^ -free HEPES-buffered solution (HBS; 10 mM HEPES, 140 mM NaCl, 4 mM KCl, 2 mM MgSO_4_, 1 mM KH_2_PO_4_, pH 7.4) and pre-incubated at 37 °C in the same solution for 60 min. Drugs were freshly prepared in HBS with or without dimethyl sulfoxide (DMSO)/1 M NaOH. Inhibitor pre-treatment was as follows: AG1024 (0.1 μM, 1 h), PTX (200 ng/ml, 14–16 h), U73122 (5 μM, 1 h), PF573228 (10 μM, 1 h), GF109203x (10 μM, 1 h) and Gö-6976 (1 μM, 1 h). Baclofen (100 μM) and CGP7930 (50 μM) were applied in time-course experiments. Baclofen (100 μM) and CGP7930 (50 μM) were applied for 10 min to detect ERK_1/2_, Akt, CREB, IGF-1R, and MARCKS phosphorylation; baclofen (100 μM), CGP7930 (50 μM), GS39783 (50 μM), or Rac BHFF (50 μM) were applied for 30 min before measuring Fmrp expression. At the end of the treatment, cells were quickly washed with ice-cold phosphate-buffered saline (PBS; pH 7.4) before lysis buffer was added to the cells, which were immediately placed on ice. The cell monolayer was scraped into Eppendorf tubes. HBS containing the same concentration of DMSO or NaOH was used as the vehicle control.

### Western blot analysis

Lysates from cultured cells were sonicated and protein concentrations were determined using the Bradford reagent (Bio-Rad Laboratories, Hertfordshire, UK). Equal amounts of protein (20 μg) were resolved by sodium dodecyl sulphate polyacrylamide gel electrophoresis. Proteins were transferred to nitrocellulose membranes (Millipore, Bedford, MA, USA), which were incubated in blocking buffer (5% non-fat dry milk in Tris-buffered saline and 0.1% Tween 20) for 1 h, followed by incubation with primary antibodies (1:1000) overnight at 4 °C and a 2 h incubation with HRP-conjugated secondary antibodies (1:20,000). Immunoreactivity was visualized on X-ray films using the enhanced chemiluminescence reagent (Pierce, Rockford, IL, USA). The density of the protein bands was measured using Image J software (National Institutes of Health, Bethesda, MD, USA) Protein ratio on the ordinates (Y) axis of the histograms was defined as the ratio between the density of each band and the sum of the densities of all the bands in a given blot.

### Reverse transcription PCR

After drug treatment, total cellular RNA was isolated using TRIzol reagent, and reverse transcription was carried out according to the manufacturer’s protocol (Invitrogen). First-strand cDNA was generated from 4 μg total RNA using oligo-dT primer and M-MLV reverse transcriptase (Invitrogen). PCR analysis was performed using the following sense and antisense primers: *Fmr1*, 5′-CCG AAC AGA TAA TCG TCC ACG-3′ and 5′-ACG CTG TCT GGC TTT TCC TTC-3′ and *β-actin*, 5′-CCG CCC TAG GCA CCA GGG TG-3′ and 5′-GGC TGG GGT GTT GAA GGT CTC AAA-3′ (internal control). The mRNA ratio was defined as the ratio between the density of each band and the sum of the densities of all bands in a given gel.

### Statistical analysis

Data are presented as mean ± SEM of at least three independent experiments. Data in Fig. 1B were analysed by the student’s t test and statistical analysis of other data was carried out with one-way ANOVA analysis.

## Additional Information

**How to cite this article**: Zhang, W. *et al.* GABA_B_ receptor upregulates fragile X mental retardation protein expression in neurons. *Sci. Rep.* **5**, 10468; doi: 10.1038/srep10468 (2015).

## Supplementary Material

Supporting Information Supplementary Figures 1-6
