# Supplementary material for: GABAB receptor upregulates fragile X mental retardation protein expression in neurons
Source: Sci Rep. 2015 May 28;5:10468. doi: 10.1038/srep10468 (PMC4447080; doi:10.1038/srep10468)
Supplement: Supporting Information — Supplementary Figures 1-6 [file srep10468-s1.pdf]

Supplemental information

GABA$_B$ receptor upregulates fragile X mental retardation protein expression in neurons

Wenhua Zhang[1,#], Chanjuan Xu[1,#,*], Haijun Tu[1,#], Yunyun Wang[1], Qian Sun[1], Ping Hu[1], Yongjian Hu[1], Philippe Rondard[2], Jianfeng Liu[1,*]

-Supplementary Figure S1

- Supplementary Figure S2
- Supplementary Figure S3
- Supplementary Figure S4
- Supplementary Figure S5
- Supplementary Figure S6
- Supplementary Figure S7
- Supplementary Figure S8
- Supplementary Figure S9
- Supplementary Figure S10
- Supplementary Figure S11
- Supplementary Figure S12

Supplemental experimental procedures

Quantitative real-time PCR (qRT-PCR) analyses

The qRT-PCR analysis was performed as previously described[1]. Briefly, CGNs were treated with baclofen or with the vehicle for indicated times, then lysed using TRIzol reagent (Invitrogen). Total RNA was prepared according to the manufacturer's instructions. The RNA concentration was determined and PCR analysis was performed using the following sense and antisense primers: *fmr1*, 5′-CCG AAC AGA TAA TCG TCC ACG-3′, antisense, 5′-ACG CTG TCT GGC TTT TCC TTC-3′ and *glyceraldehyde-3-phosphate dehydrogenase*, 5′-TCG GAG TCA ACG GAT TTG GTC GT-3′ and 5′-TGCCATGGGTGGAATCATATTGGA-3′ (used as a reference gene).

RNA interference experiments

Mammalian shRNA constructs were designed as previously described[3]. The shRNA targeted bases 3476 to 3494 (NM_000875.2) of the IGF-1R cDNA; the forward and reverse DNA templates were gatccccgaagatttcacagtcaacttcctgtcagattgactgtgaaatcttcggtttttg and aattcaaaaaccgaagatttcacagtcaatctgacaggaagttgactgtgaaatcttcggg, respectively. The shRNA oligonucleotides were phosphorylated, annealed, and then ligated into linearized pSIH-H1-copGFP shRNA vector (System Biosciences, Mountain View, CA, USA), then digested with *Eco*RI/*Bam*HI. MEFs were transfected with shRNA plasmid using Lipofectamine 2000 reagent (Invitrogen) and then with HA-$GABA_{B1}$ and Flag-$GABA_{B2}$ plasmids 24 h later before drug treatment.

Drug treatments

Cultures were washed once with HBS (HEPES-buffered solution, 10 mM HEPES, 140 mM NaCl, 4 mM KCl, 2 mM $MgSO_4$, 1 mM KH_2PO_4, Ca^{2+} free, pH 7.4) and pre-incubated at 37 ℃ with HBS for 60 min. Cells were treated by adding freshly made drugs to HBS. Inhibitors were pretreated in cells as followed: CGP54626 (10 μM, 20min), Gö-6983 (2 μM, 1h). Baclofen (100 μM) and CGP7930 (50 μM) were applied for 10 min for $ERK_{1/2}$, Akt, CREB, IGF-1R and MARCKS phosphorylation detection. For Fmrp expression measurement, baclofen (100 μM), CGP7930 (50 μM), GS39783 (50 μM) or Rac BHFF (50 μM) were applied for 30 min. At the end of the treatment, cells were washed quickly with ice-cold PBS (pH 7.4), then lysis buffer was added to the cells and placed immediately on ice. The cell monolayer was scraped into Eppendorf tubes. Drugs were dissolved in HBS with or without DMSO/1M NaOH. Whenever DMSO/NaOH were used, HBS containing the same concentration of DMSO or NaOH were used as the control vehicle.

Supplemental data figure legends

Figure S1

Activation of $GABA_B$ receptor upregulates Fmrp expression in CGNs. Full-length images of the cropped blot shown in Figure 1.

Figure S2

Activation of $GABA_B$ receptor upregulates *Fmr1* mRNA expression in CGNs. *Fmr1* mRNA expression measured upon treatment with baclofen.

Figure S3

$GABA_B$ receptor-induced CREB signaling is required for Fmrp upregulation. Full-length images of the cropped blot shown in Figure 2.

Figure S4

Activation of CREB by $GABA_B$ receptor is through the $GABA_{B2}$ subunit. (A) CGNs were treated with CGP54626 prior to treatment with baclofen or CGP7930. Phosphorylated and total CREB levels were measured by western blotting, and pCREB ratio was defined as in Figure 2B. Data are representative of three independent experiments. ***P < 0.001 vs. basal levels. $^{###}$P < 0.001 vs. baclofen-treated cells; ns, not significant vs CGP7930-treated cells. **(B)** Full-length images of the cropped blot shown in panel A.

Figure S5

$GABA_B$ receptor-mediated CREB phosphorylation is dependent on $G\alpha_{i/o}$ protein, PLCβ and FAK. Full-length images of the cropped blot shown in Figure 3.

Figure S6

$GABA_B$ receptor-mediated transactivation of IGF-1R is required for CREB phosphorylation. Full-length images of the cropped blot shown in Figure 4.

Figure S7

Role of IGF-1R in $GABA_B$ receptor-induced CREB activation. (A) MEFs were transfected with shRNAs targeting $GABA_B$ receptor and IGF-1R, then treated with baclofen. $ERK_{1/2}$ and CREB phosphorylation levels were measured. **(B)** Full-length images of the cropped blot shown in panel A.

Figure S8

PKC activated by $GABA_B$ receptor acts downstream of FAK and is independent of IGF-1R signaling. Full-length images of the cropped blot shown in Figure 5.

Figure S9

PKC is required for $GABA_B$ receptor-induced CREB activation. Full-length images of the cropped blot shown in Figure 6.

Figure S10

Role of PKC in $GABA_B$ receptor-activated $ERK_{1/2}$, Akt, and CREB phosphorylation. (A) CGNs were pretreated with Gö-6983, followed by treatment with baclofen. Akt, $ERK_{1/2}$, and CREB phosphorylation levels were quantified by western blotting, and the protein ratio was defined as the ratio between the density of each band and the sum of the densities of all the bands in a given blot. Data represent the mean ± SEM from three independent experiments. ***$P < 0.001$ vs. basal level. $^{\#\#\#}P < 0.001$; ns, not significant vs. baclofen treated group. **(B)** MEFs co-transfected with $GABA_{B1}$ and $GABA_{B2}$ were pretreated with control or PKCβII siRNA, followed by treatment with baclofen. PKCβII, $pERK_{1/2}$, and pCREB levels were analyzed by western blotting.

Figure S11

IGF-1R and PKC are involved in $GABA_B$ receptor-mediated Fmrp synthesis. Full-length images of the cropped blot shown in Figure 7.

Figure S12

Effect of $GABA_B$ receptor PAMs on Akt and CREB activation. (A) CGNs were treated with vehicle, baclofen, CGP7930, GS39783, or Rac BHFF, and the levels of pAkt and pCREB and total protein were measured by western blotting. The protein ratio was defined as the ratio between the density of each band and the sum of the densities of all the bands in a given blot. Data represent the mean ± SEM from three independent experiments. ***P < 0.001; ns, not significant vs. basal level. **(B)** Full-length images of the cropped blot shown in panel A.

References

1. Li, B. et al. FGF-2 prevents cancer cells from ER stress-mediated apoptosis via enhancing proteasome-mediated Nck degradation. *Biochem J* **452**, 139-45 (2013).
2. Maurel, D. et al. Cell-surface protein-protein interaction analysis with time-resolved FRET and snap-tag technologies: application to GPCR oligomerization. *Nat Methods* **5**, 561-7 (2008).
3. Dong, A.Q., Kong, M.J., Ma, Z.Y., Qian, J.F. & Xu, X.H. Down-regulation of IGF-IR using small, interfering, hairpin RNA (siRNA) inhibits growth of human lung cancer cell line A549 in vitro and in nude mice. *Cell Biol Int* **31**, 500-7 (2007).

Figure S1

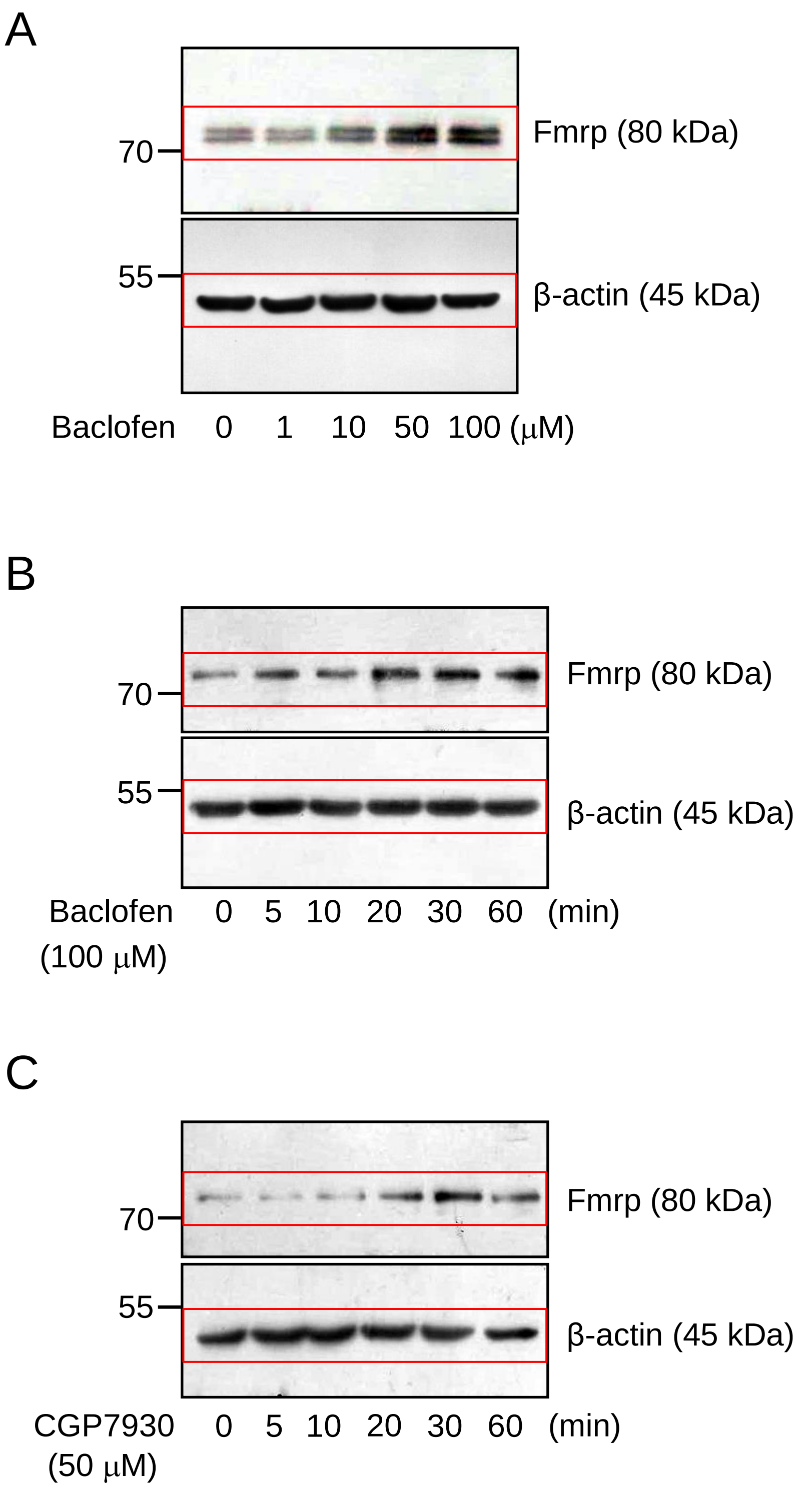

A
70
55
Fmrp (80 kDa)
β-actin (45 kDa)
Baclofen 0 1 10 50 100 (μM)
B
70
55
Fmrp (80 kDa)
β-actin (45 kDa)
Baclofen (100 μM) 0 5 10 20 30 60 (min)
C
70
55
Fmrp (80 kDa)
β-actin (45 kDa)
CGP7930 (50 μM) 0 5 10 20 30 60 (min)

Figure S2

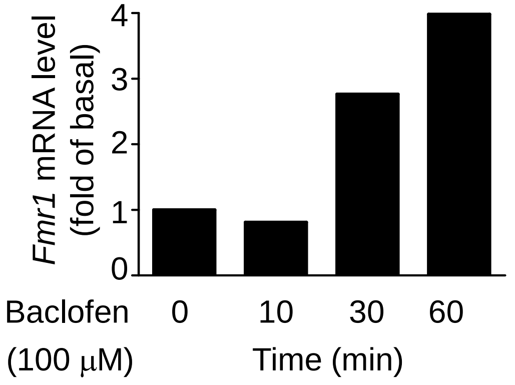

Fmr1 mRNA level
(fold of basal)
0
1
2
3
4
Baclofen
(100 μM)
0
10
30
60
Time (min)

Figure S3

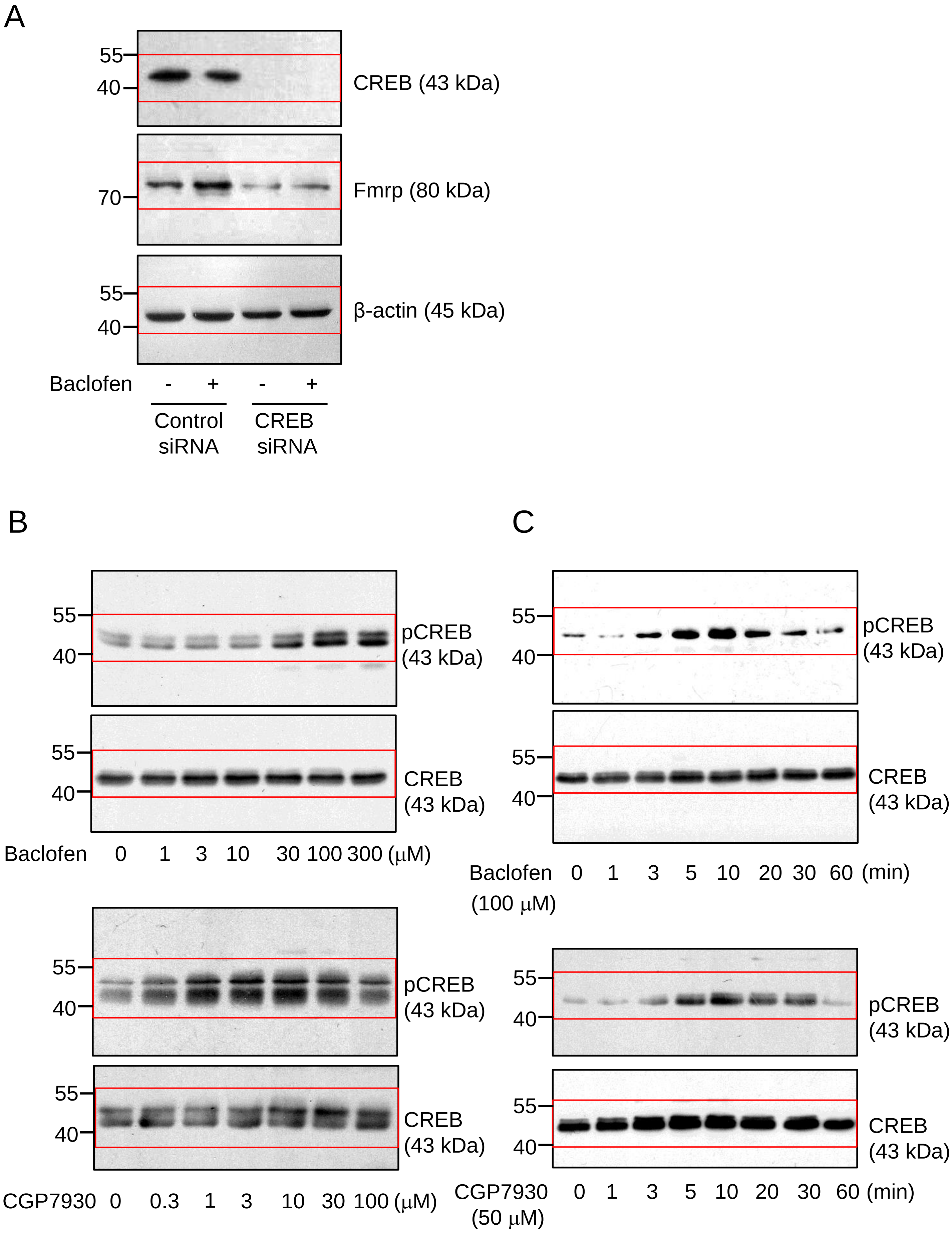

A
55
40
CREB (43 kDa)
70
Fmrp (80 kDa)
55
40
β-actin (45 kDa)
Baclofen - + - +
Control siRNA
CREB siRNA
B
55
40
pCREB (43 kDa)
55
40
CREB (43 kDa)
Baclofen 0 1 3 10 30 100 300 (μM)
55
40
pCREB (43 kDa)
55
40
CREB (43 kDa)
CGP7930 0 0.3 1 3 10 30 100 (μM)
C
55
40
pCREB (43 kDa)
55
40
CREB (43 kDa)
Baclofen (100 μM) 0 1 3 5 10 20 30 60 (min)
55
40
pCREB (43 kDa)
55
40
CREB (43 kDa)
CGP7930 (50 μM) 0 1 3 5 10 20 30 60 (min)

Figure S4

A

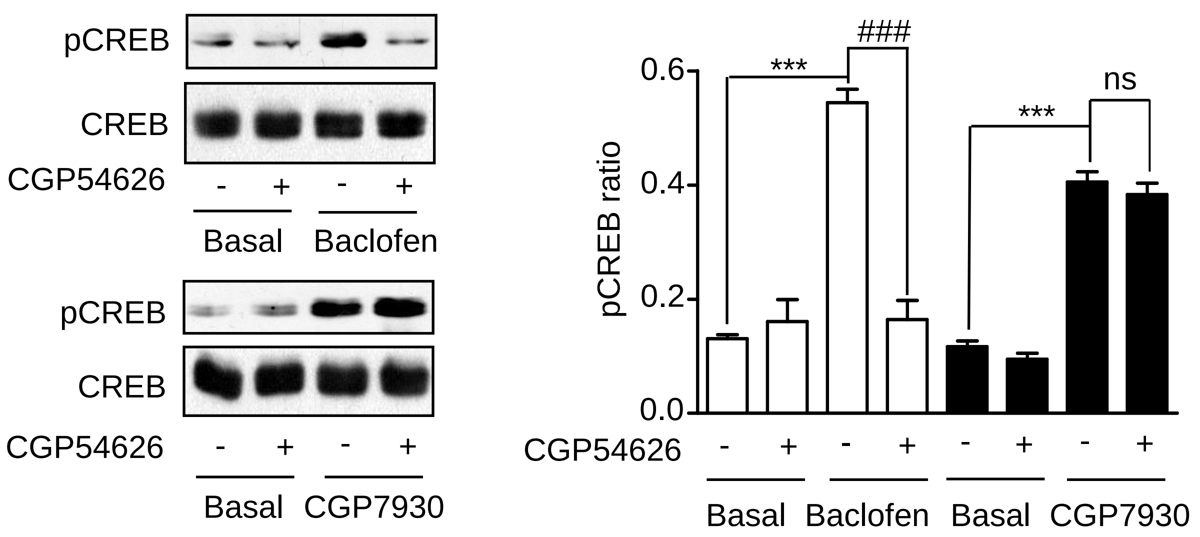

pCREB
CREB
CGP54626
- + - +
Basal
Baclofen
pCREB
CREB
CGP54626
- + - +
Basal
CGP7930
pCREB ratio
0.0
0.2
0.4
0.6
***
###
***
ns
CGP54626
- + - + - + - +
Basal
Baclofen
Basal
CGP7930

B

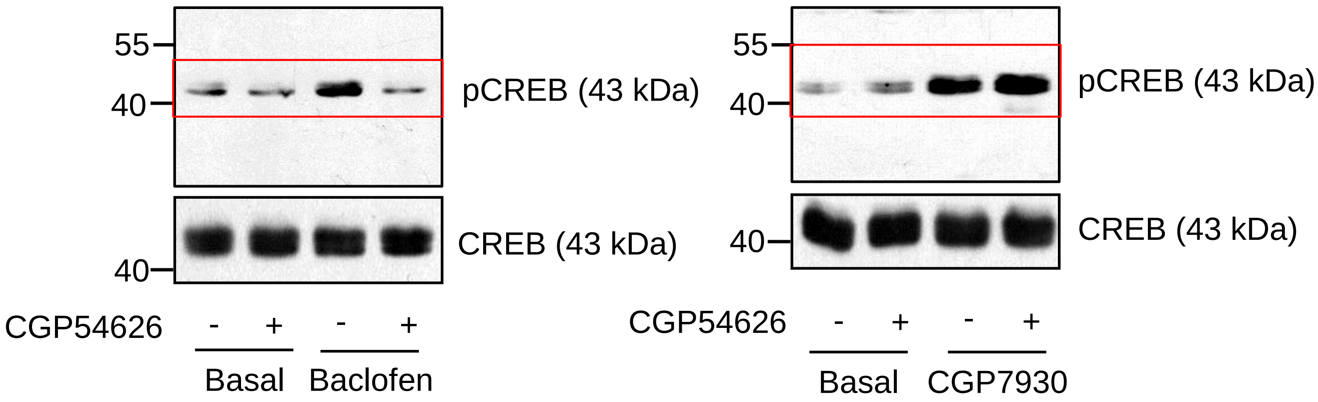

55
40
40
pCREB (43 kDa)
CREB (43 kDa)
CGP54626
- + - +
Basal
Baclofen
55
40
40
pCREB (43 kDa)
CREB (43 kDa)
CGP54626
- + - +
Basal
CGP7930

Figure S5

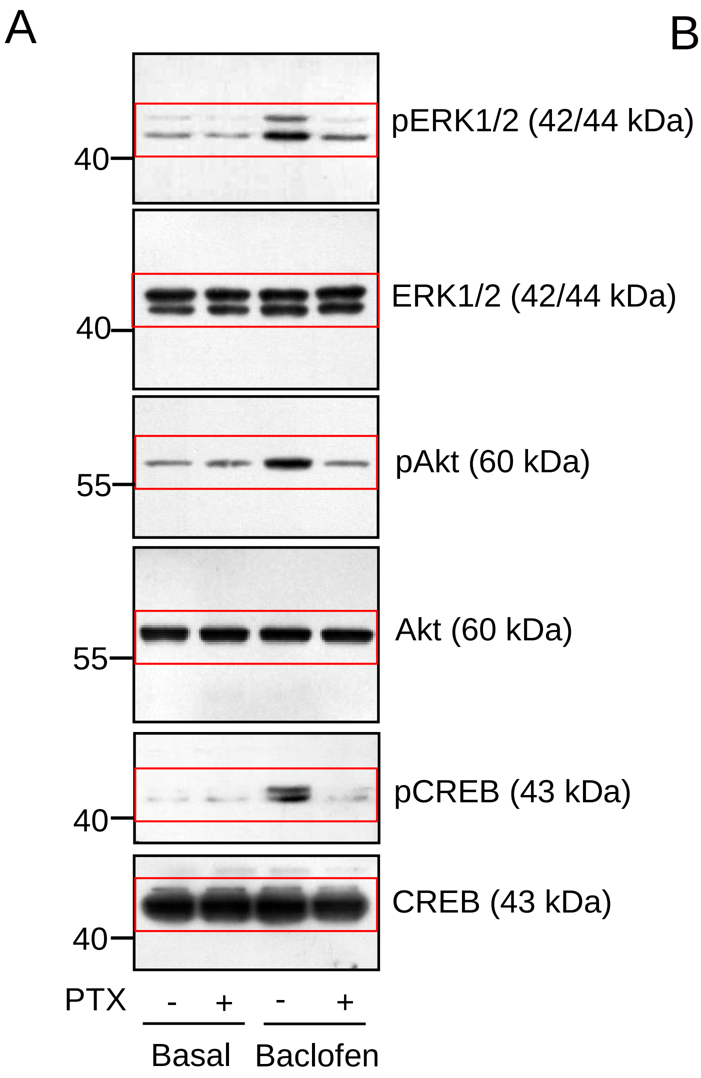
A
pERK1/2 (42/44 kDa)
ERK1/2 (42/44 kDa)
pAkt (60 kDa)
Akt (60 kDa)
pCREB (43 kDa)
CREB (43 kDa)
40
40
55
55
40
40
PTX
- + - +
Basal
Baclofen

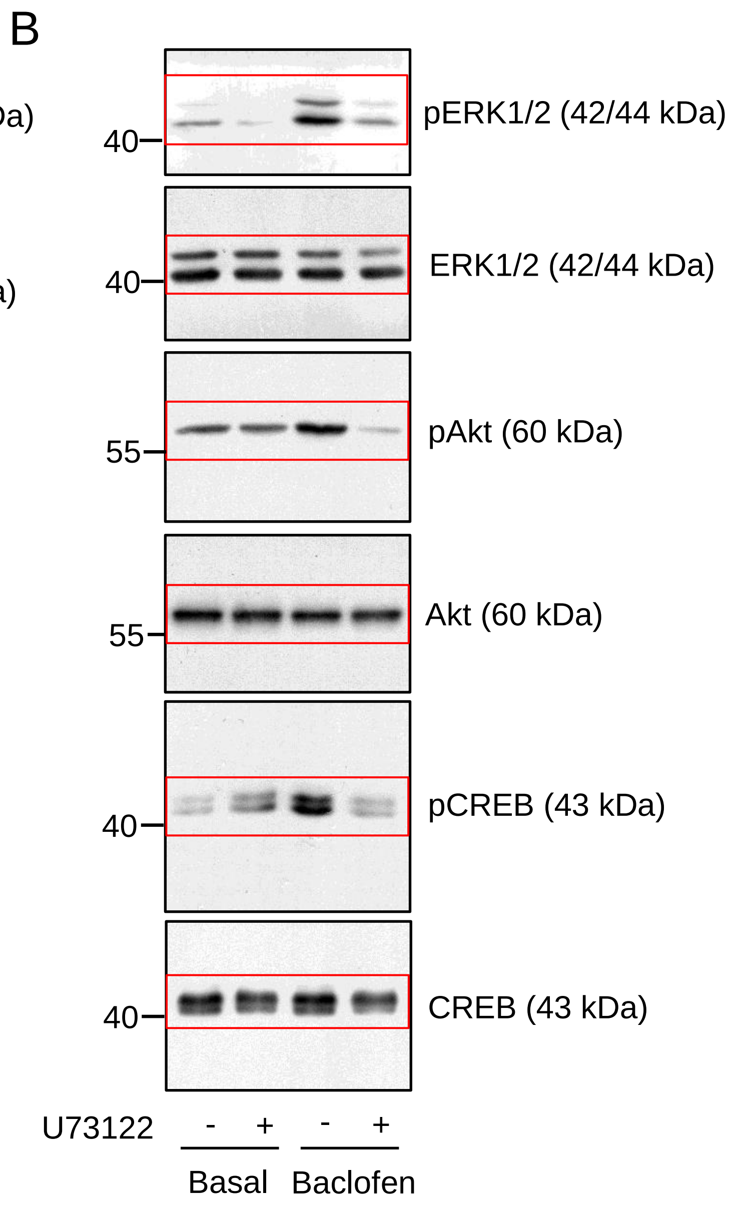
B
pERK1/2 (42/44 kDa)
ERK1/2 (42/44 kDa)
pAkt (60 kDa)
Akt (60 kDa)
pCREB (43 kDa)
CREB (43 kDa)
40
40
55
55
40
40
U73122
- + - +
Basal
Baclofen

Figure S5

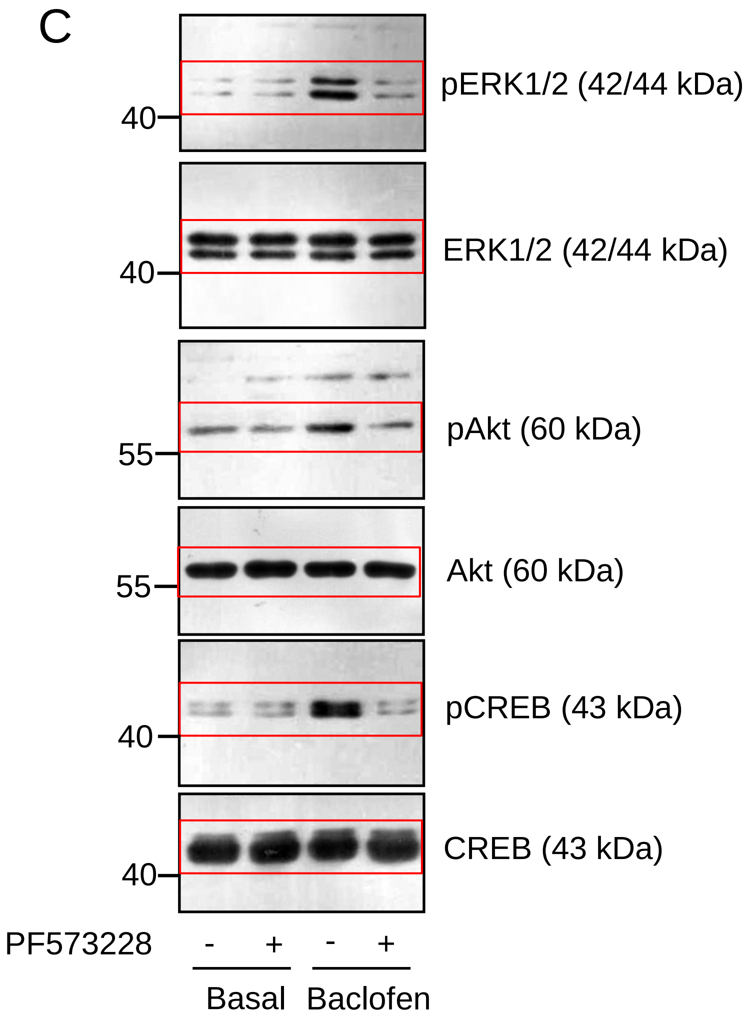
C
40
pERK1/2 (42/44 kDa)
40
ERK1/2 (42/44 kDa)
55
pAkt (60 kDa)
55
Akt (60 kDa)
40
pCREB (43 kDa)
40
CREB (43 kDa)
PF573228
-
+
-
+
Basal
Baclofen

Figure S6

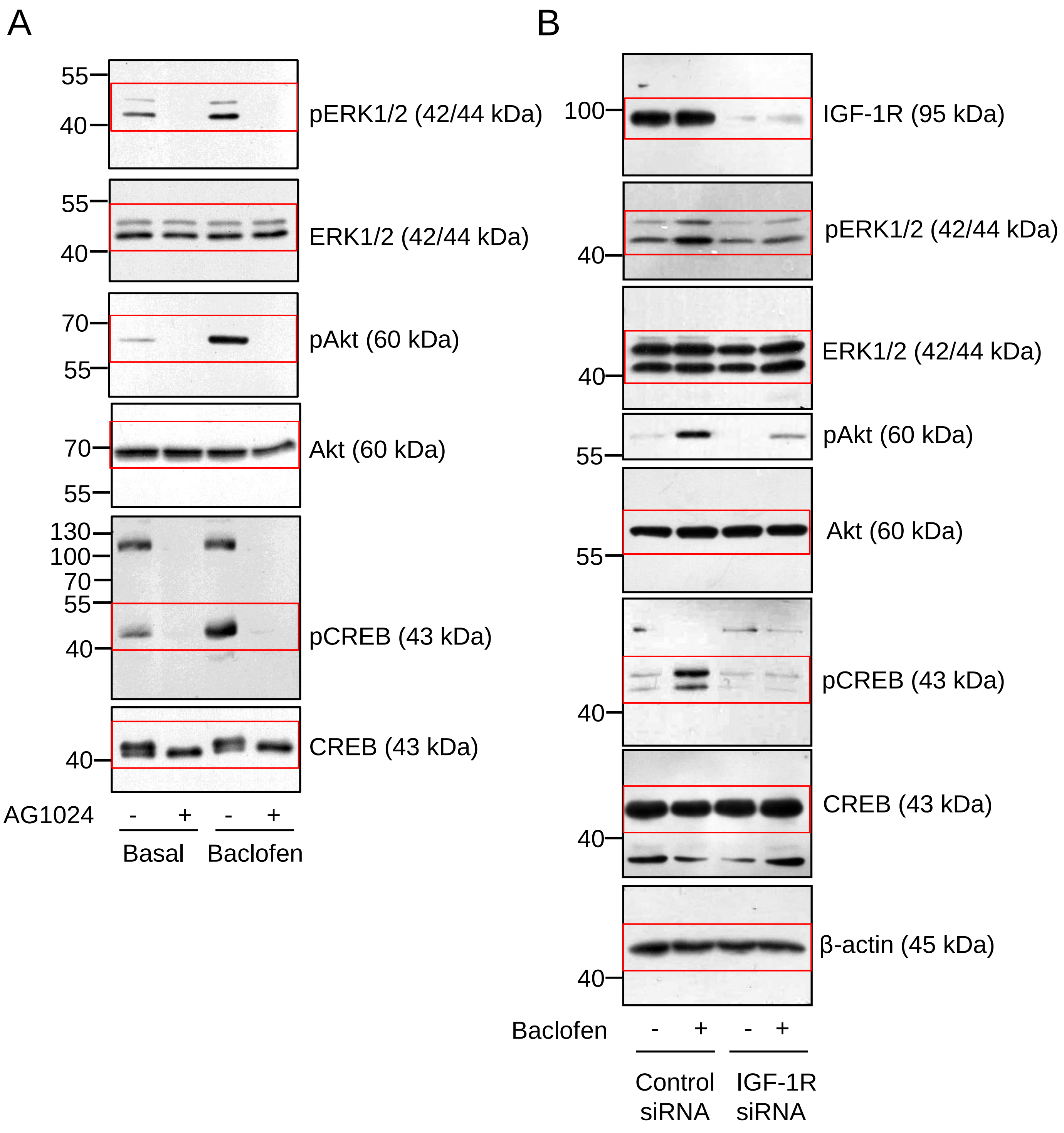
A
55
40
pERK1/2 (42/44 kDa)
55
40
ERK1/2 (42/44 kDa)
70
55
pAkt (60 kDa)
70
55
Akt (60 kDa)
130
100
70
55
40
pCREB (43 kDa)
40
CREB (43 kDa)
AG1024 - + - +
Basal
Baclofen
B
100
IGF-1R (95 kDa)
40
pERK1/2 (42/44 kDa)
40
ERK1/2 (42/44 kDa)
55
pAkt (60 kDa)
55
Akt (60 kDa)
40
pCREB (43 kDa)
40
CREB (43 kDa)
40
β-actin (45 kDa)
Baclofen - + - +
Control siRNA
IGF-1R siRNA

Figure S7

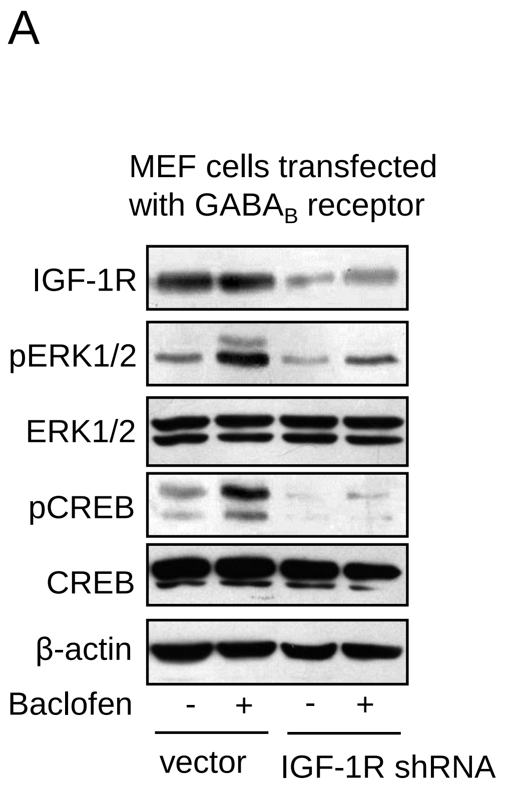
A
MEF cells transfected
with $GABA_B$ receptor
IGF-1R
pERK1/2
ERK1/2
pCREB
CREB
β-actin
Baclofen
- + - +
vector
IGF-1R shRNA

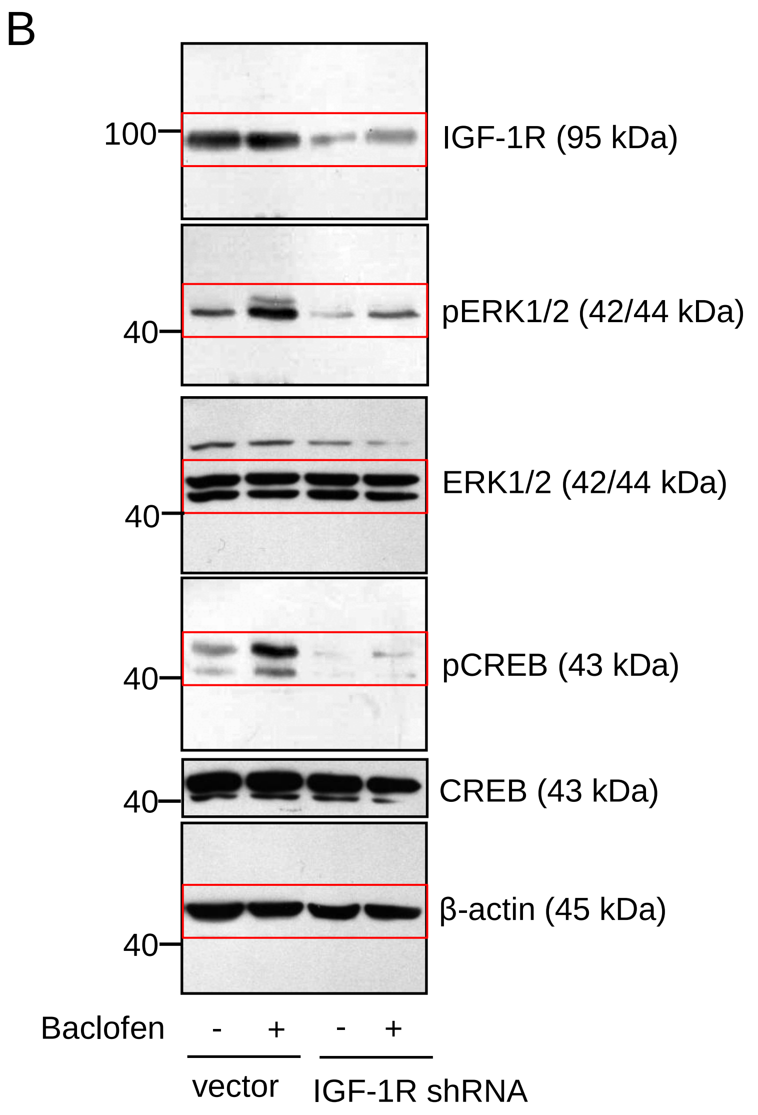
B
100
40
40
40
40
40
IGF-1R (95 kDa)
pERK1/2 (42/44 kDa)
ERK1/2 (42/44 kDa)
pCREB (43 kDa)
CREB (43 kDa)
β-actin (45 kDa)
Baclofen
- + - +
vector
IGF-1R shRNA

Figure S8

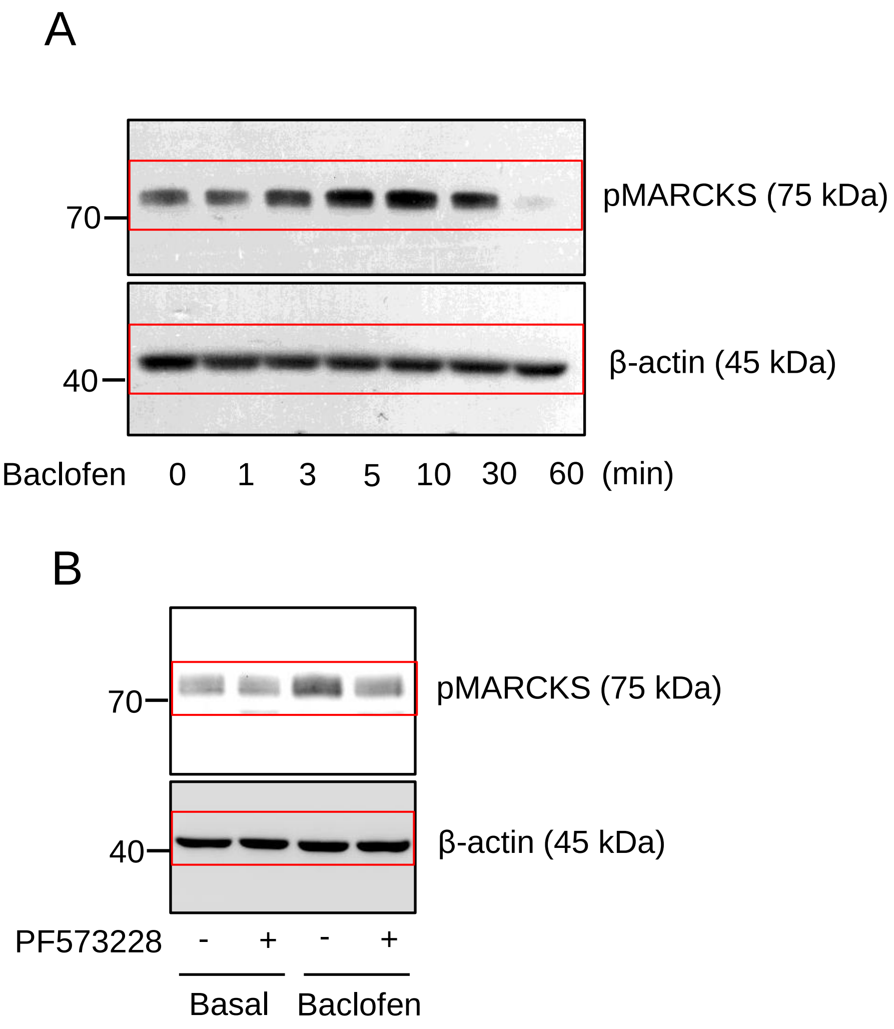
A
70
40
pMARCKS (75 kDa)
β-actin (45 kDa)
Baclofen 0 1 3 5 10 30 60 (min)
B
70
40
pMARCKS (75 kDa)
β-actin (45 kDa)
PF573228 - + - +
Basal
Baclofen

Figure S8

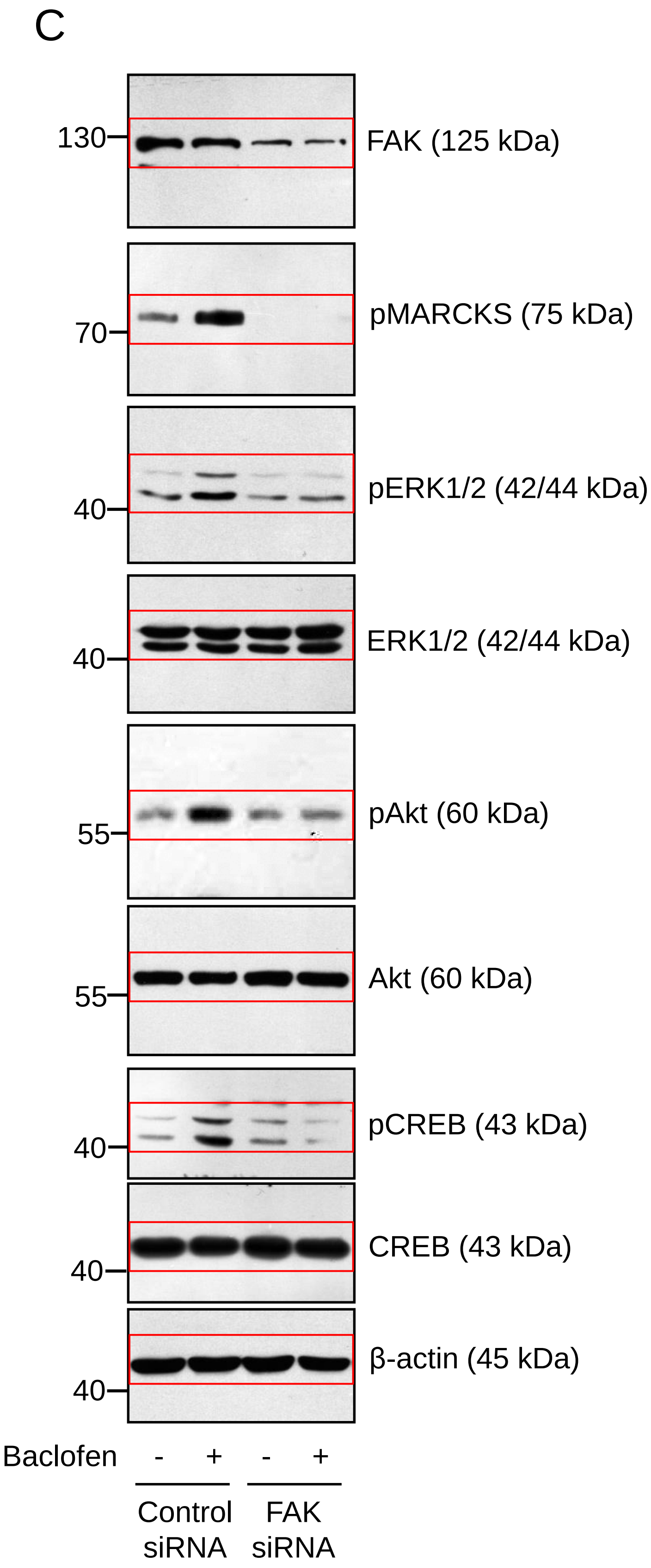
C
130
FAK (125 kDa)
70
pMARCKS (75 kDa)
40
pERK1/2 (42/44 kDa)
40
ERK1/2 (42/44 kDa)
55
pAkt (60 kDa)
55
Akt (60 kDa)
40
pCREB (43 kDa)
40
CREB (43 kDa)
40
β-actin (45 kDa)
Baclofen - + - +
Control siRNA
FAK siRNA

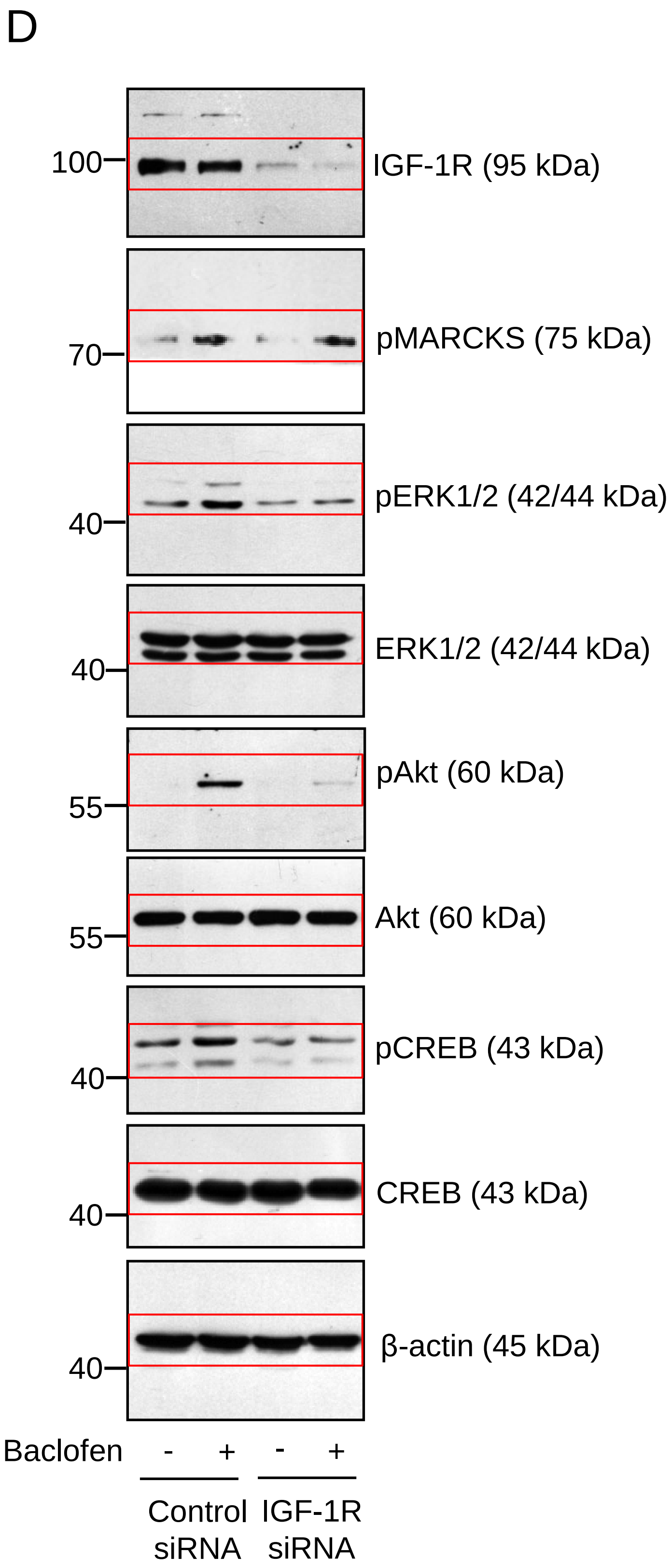
D
100
IGF-1R (95 kDa)
70
pMARCKS (75 kDa)
40
pERK1/2 (42/44 kDa)
40
ERK1/2 (42/44 kDa)
55
pAkt (60 kDa)
55
Akt (60 kDa)
40
pCREB (43 kDa)
40
CREB (43 kDa)
40
β-actin (45 kDa)
Baclofen - + - +
Control siRNA
IGF-1R siRNA

Figure S9

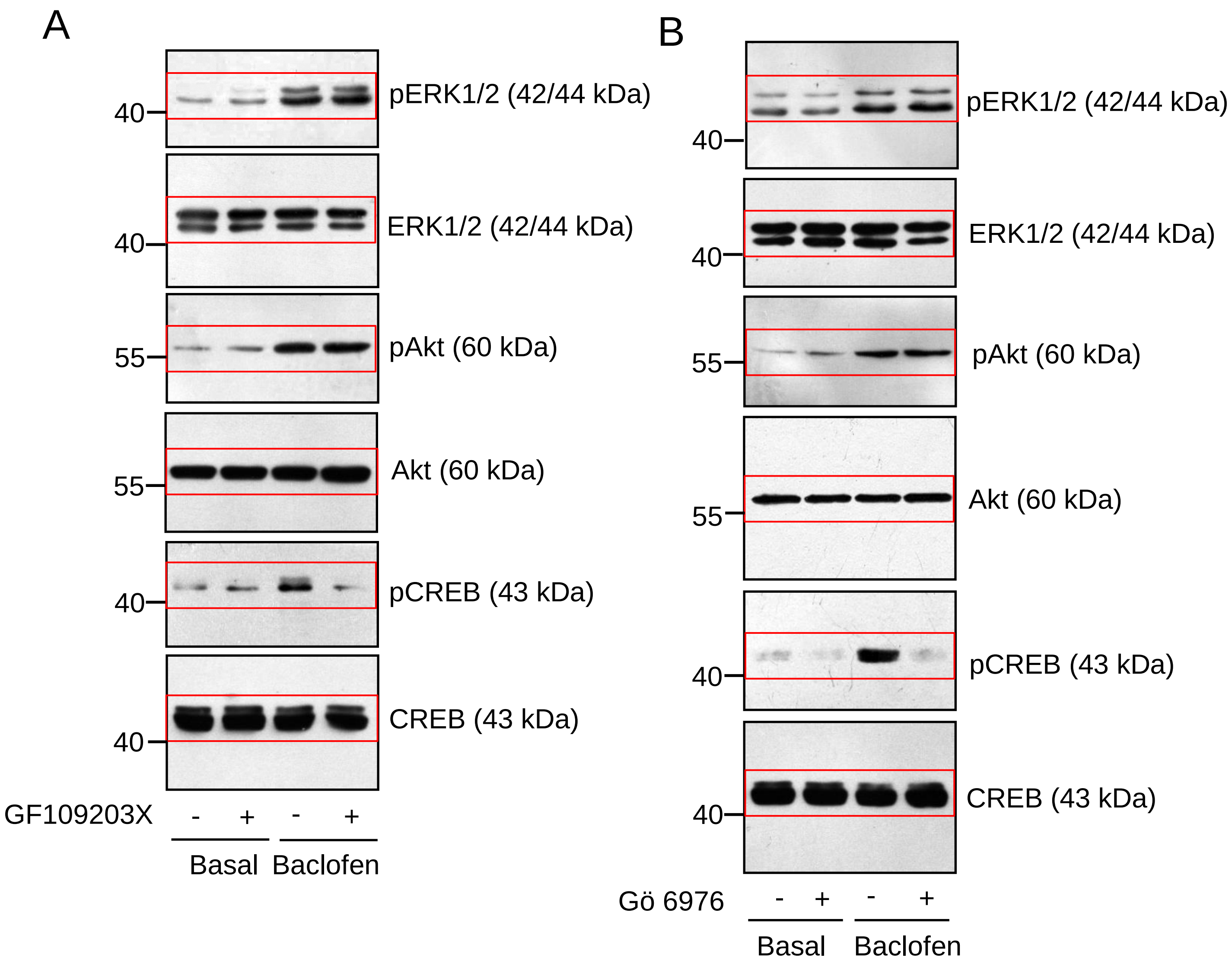
A
pERK1/2 (42/44 kDa)
ERK1/2 (42/44 kDa)
pAkt (60 kDa)
Akt (60 kDa)
pCREB (43 kDa)
CREB (43 kDa)
40
55
GF109203X
- + - +
Basal
Baclofen
B
pERK1/2 (42/44 kDa)
ERK1/2 (42/44 kDa)
pAkt (60 kDa)
Akt (60 kDa)
pCREB (43 kDa)
CREB (43 kDa)
Gö 6976
- + - +
Basal
Baclofen

Figure S9

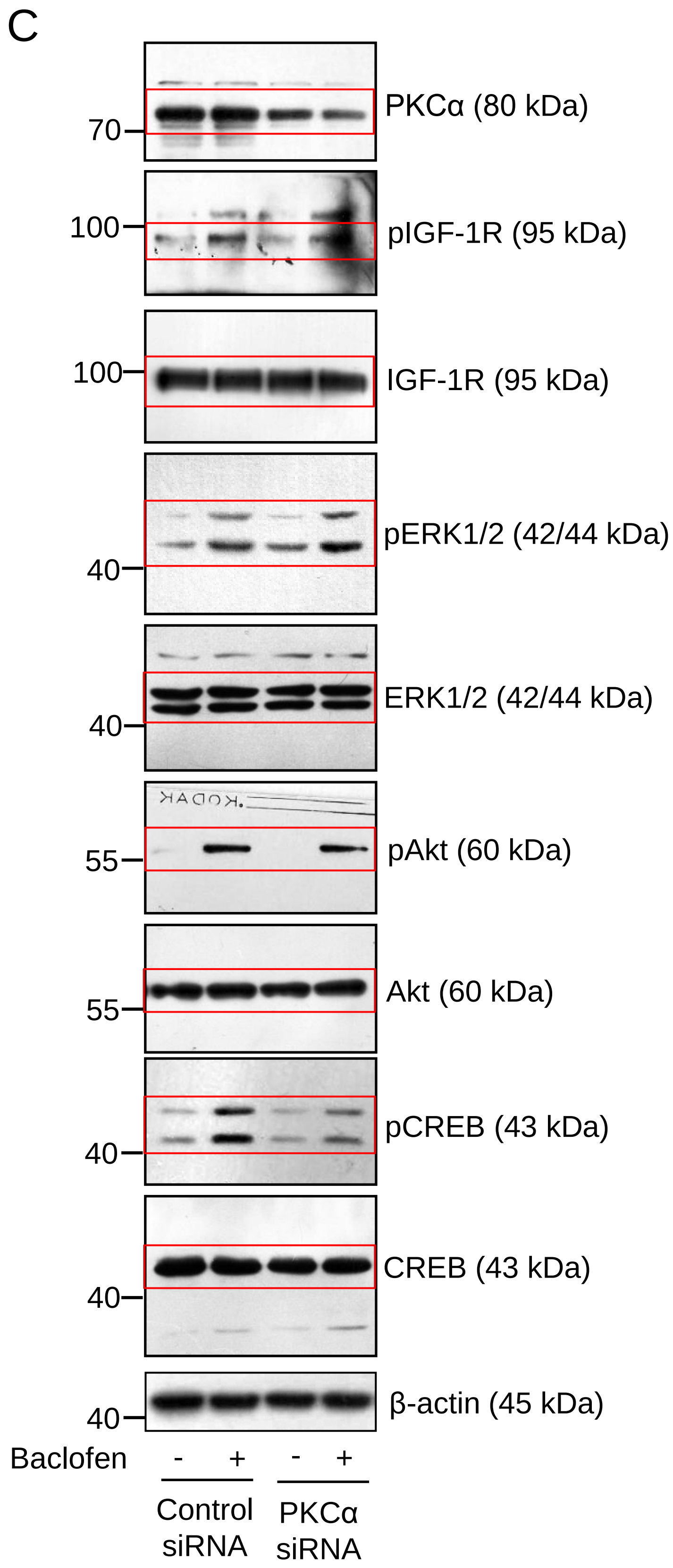
C
PKCα (80 kDa)
70
pIGF-1R (95 kDa)
100
IGF-1R (95 kDa)
100
pERK1/2 (42/44 kDa)
40
ERK1/2 (42/44 kDa)
40
pAkt (60 kDa)
55
Akt (60 kDa)
55
pCREB (43 kDa)
40
CREB (43 kDa)
40
β-actin (45 kDa)
40
Baclofen
- + - +
Control siRNA
PKCα siRNA

Figure S10

A

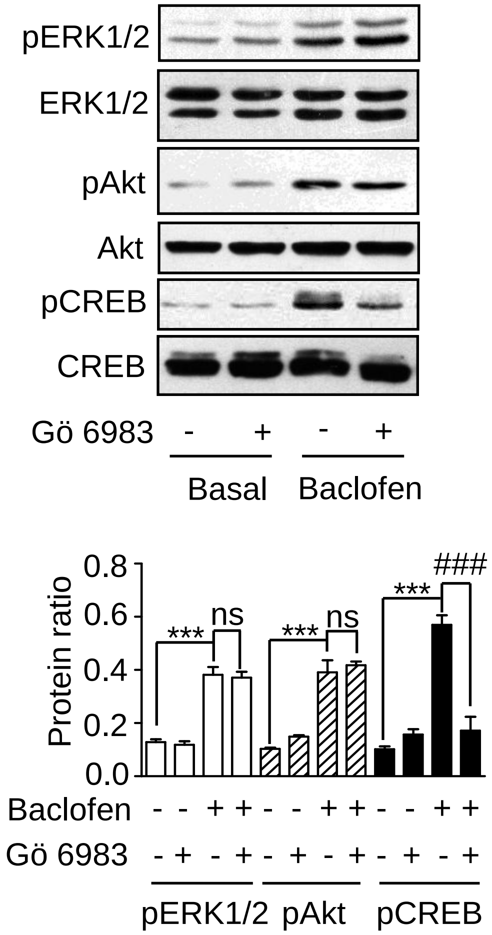

B

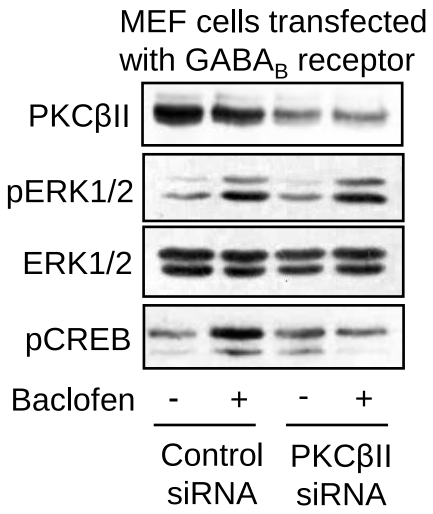

Figure S11

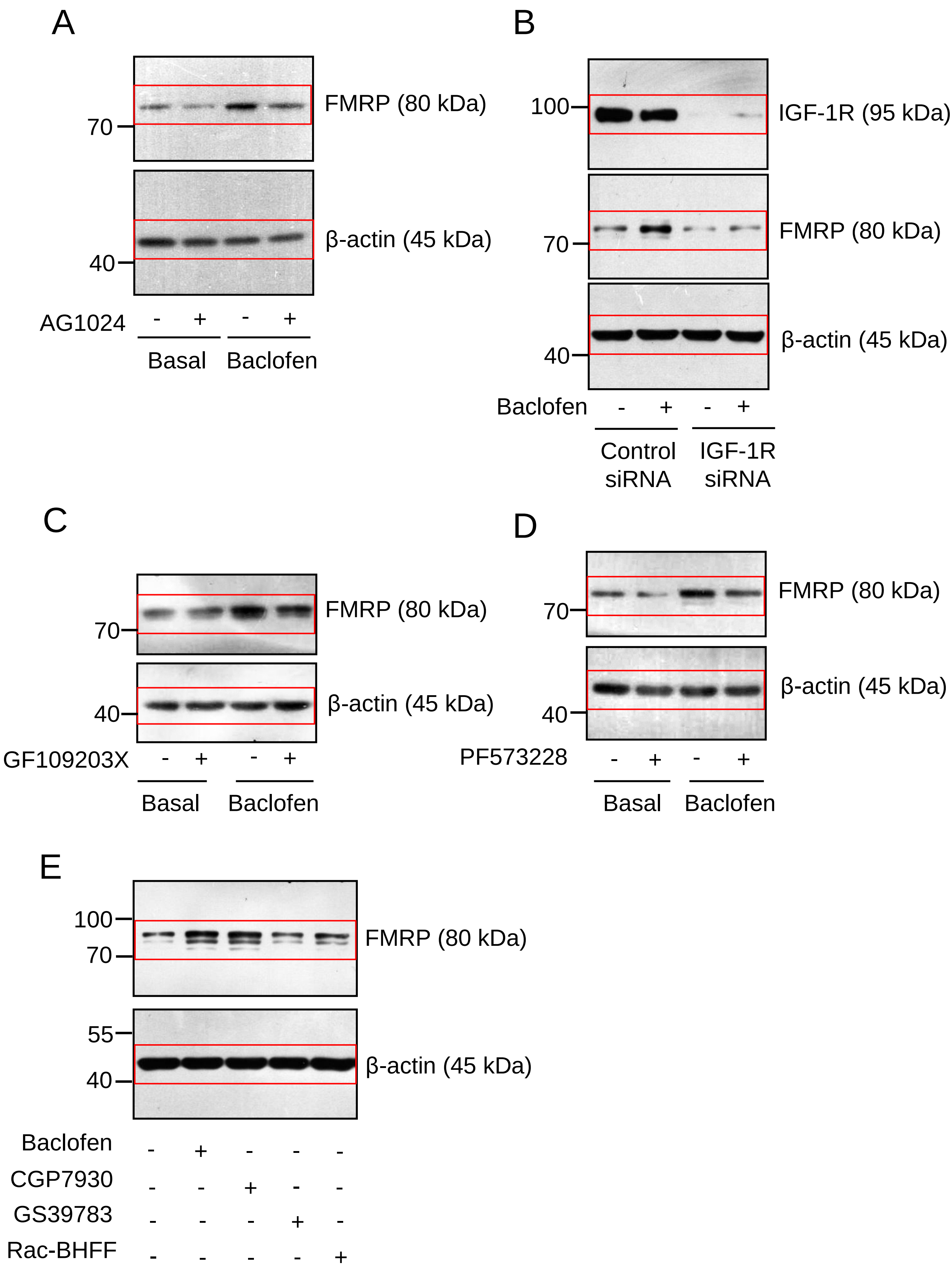
A
70
40
FMRP (80 kDa)
β-actin (45 kDa)
AG1024 - + - +
Basal
Baclofen
B
100
70
40
IGF-1R (95 kDa)
FMRP (80 kDa)
β-actin (45 kDa)
Baclofen - + - +
Control siRNA
IGF-1R siRNA
C
70
40
FMRP (80 kDa)
β-actin (45 kDa)
GF109203X - + - +
Basal
Baclofen
D
70
40
FMRP (80 kDa)
β-actin (45 kDa)
PF573228 - + - +
Basal
Baclofen
E
100
70
55
40
FMRP (80 kDa)
β-actin (45 kDa)
Baclofen - + - - -
CGP7930 - - + - -
GS39783 - - - + -
Rac-BHFF - - - - +

Figure S12

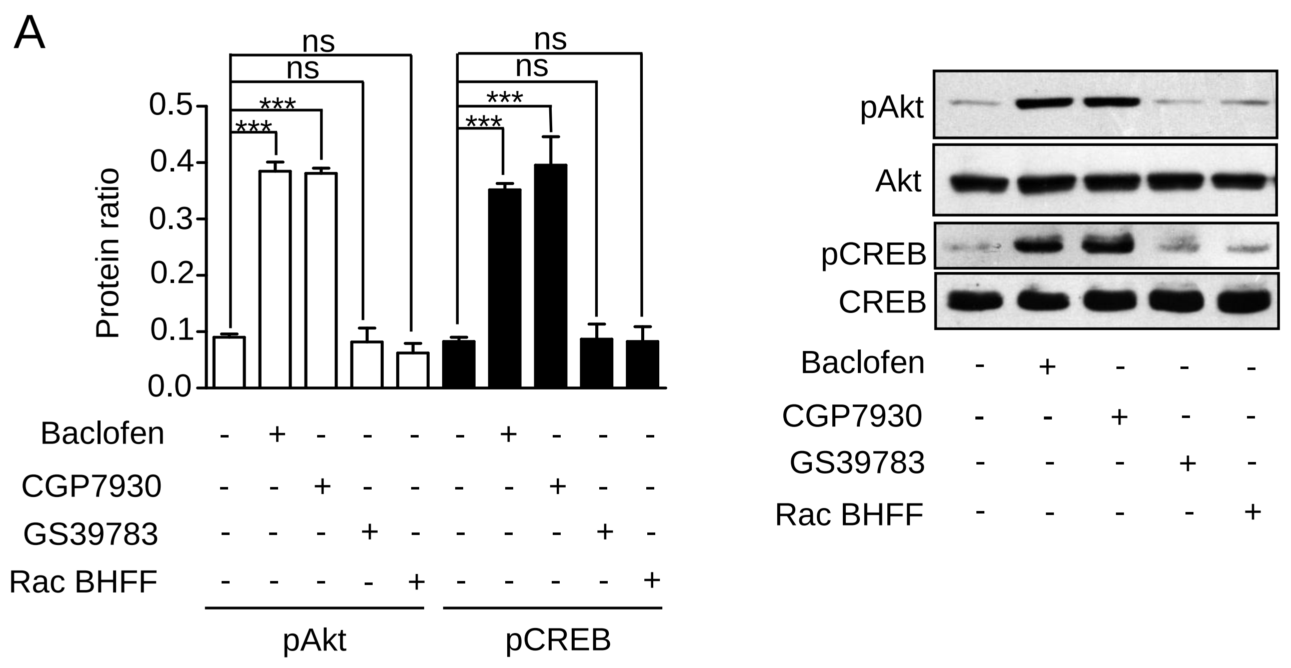
A
ns
ns
***
***
ns
ns
***
***
0.5
0.4
0.3
0.2
0.1
0.0
Protein ratio
Baclofen - + - - - - + - - -
CGP7930 - - + - - - - + - -
GS39783 - - - + - - - - + -
Rac BHFF - - - - + - - - - +
pAkt
pCREB
pAkt
Akt
pCREB
CREB
Baclofen - + - - -
CGP7930 - - + - -
GS39783 - - - + -
Rac BHFF - - - - +

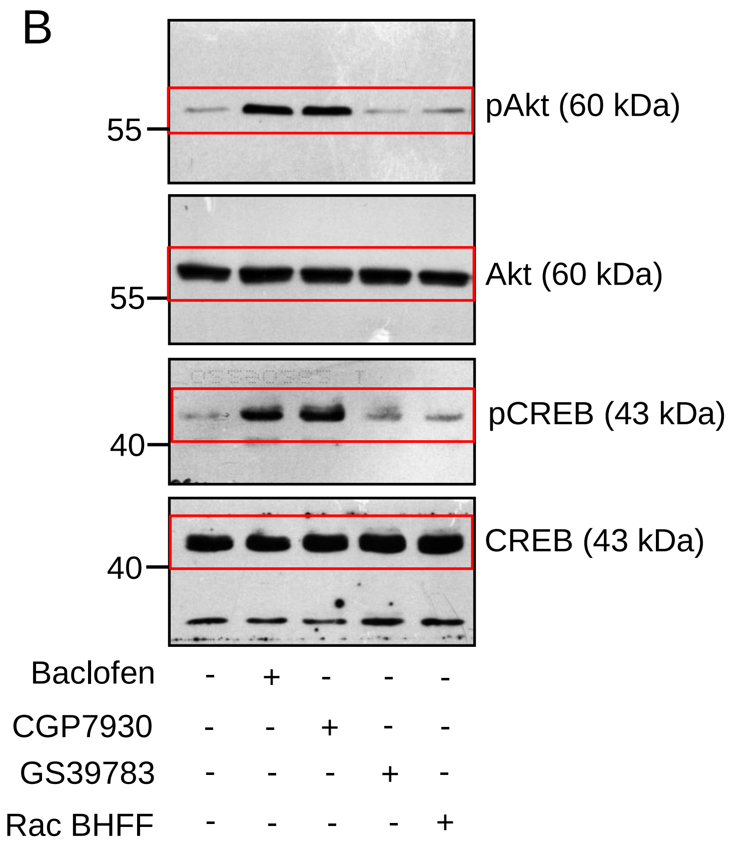
B
55
pAkt (60 kDa)
55
Akt (60 kDa)
40
pCREB (43 kDa)
40
CREB (43 kDa)
Baclofen - + - - -
CGP7930 - - + - -
GS39783 - - - + -
Rac BHFF - - - - +
